# Tnfrsf4-expressing regulatory T cells promote immune escape of chronic myeloid leukemia stem cells

**DOI:** 10.1172/jci.insight.151797

**Published:** 2021-12-08

**Authors:** Magdalena Hinterbrandner, Viviana Rubino, Carina Stoll, Stefan Forster, Noah Schnüriger, Ramin Radpour, Gabriela M. Baerlocher, Adrian F. Ochsenbein, Carsten Riether

**Affiliations:** 1 Department of Medical Oncology, Inselspital, Bern University Hospital,; 2 Department for BioMedical Research (DBMR),; 3 Graduate School of Cellular and Biomedical Sciences, and; 4 Department of Hematology and Central Hematology Laboratory, Inselspital, Bern University Hospital, University of Bern, Switzerland.

**Keywords:** Hematology, Stem cells, Cancer immunotherapy, Leukemias

## Abstract

Leukemia stem cells (LSCs) promote the disease and seem resistant to therapy and immune control. Why LSCs are selectively resistant against elimination by CD8^+^ cytotoxic T cells (CTLs) is still unknown. In this study, we demonstrate that LSCs in chronic myeloid leukemia (CML) can be recognized and killed by CD8^+^ CTLs in vitro. However, Tregs, which preferentially localized close to CD8^+^ CTLs in CML BM, protected LSCs from MHC class I–dependent CD8^+^ CTL–mediated elimination in vivo. BM Tregs in CML were characterized by the selective expression of tumor necrosis factor receptor 4 (Tnfrsf4). Stimulation of Tnfrsf4 signaling did not deplete Tregs but reduced the capacity of Tregs to protect LSCs from CD8^+^ CTL–mediated killing. In the BM of newly diagnosed CML patients, *TNFRSF4* mRNA levels were significantly increased and correlated with the expression of the Treg-restricted transcription factor *FOXP3*. Overall, these results identify Tregs as key regulators of immune escape of LSCs and TNFRSF4 as a potential target to reduce the function of Tregs and boost antileukemic immunity in CML.

## Graphical abstract

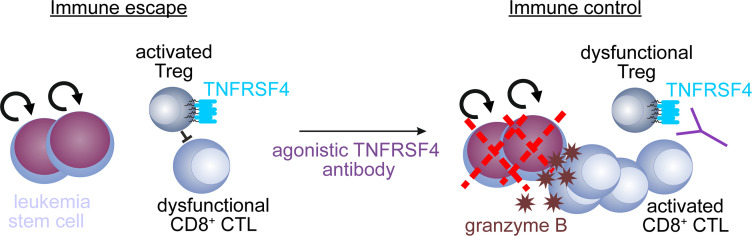

## Introduction

BCR-ABL1 Tyrosine kinase inhibitors (TKI) have revolutionized the clinical management of chronic myeloid leukemia (CML) patients. These TKIs remarkably improved the prognosis of CML patients, as indicated by the induction of durable complete cytogenetic hematologic responses in the majority of patients and even deep molecular remissions in a proportion of patients (1–3). Only half of the latter patients can permanently discontinue TKI therapy and maintain a treatment-free remission (4). This is due to the insufficient action of TKIs on quiescent, self-renewing leukemia stem cells (LSCs) in the BM of the patients. Such persistent LSCs can maintain the disease and are responsible for relapse of the disease upon drug discontinuation (5).

Immunotherapy may be a potential approach to eradicate such TKI-insensitive cells/LSCs in CML patients. Leukemia cells, including LSCs, are sensitive to lysis by T cells and NK cells. The relevance of allo-reactive CD8^+^ T cells in the control of leukemia has impressively been documented in leukemia patients receiving allogenic hematopoietic stem cell transplantation (aHSCT; refs. 6–8) or in patients receiving donor lymphocyte infusions after relapse (7, 9, 10). However, the contribution of the endogenous adaptive immune system to the pathophysiology of leukemia is less evident. Recent studies using highly sensitive detection methods for the BCR-ABL1 transcript demonstrated that residual leukemic cells and CML LSCs can be detected even in patients who are in a molecular remission after TKI discontinuation (11, 12). These findings suggest that the host immune system may contribute to the control of these residual cells and prevent CML progression/relapse in these patients.

Compared with the majority of solid tumors, CML cells have a low mutational burden resulting in the generation of only a limited number of neo-antigens that may be detected by specific CD8^+^ cytotoxic T cells (CTLs; ref. 13). As yet, endogenous CD4^+^ and CD8^+^ T cell responses directed against leukemia-specific antigens (LSA) and leukemia-associated antigens have been detected in chronic phase CML patients (14–17) — particularly LSA derived from the junctional region of BCR-ABL1, which represent CML-specific neo-antigens. Furthermore, aberrantly expressed self-proteins such as Wilms’ tumor protein-1 (WT-1), Proteinase 3 (PR 3), and human telomerase reverse transcriptase (hTERT) have also been shown to be immunogenic and to elicit specific T cell responses in vitro and in vivo (14, 16, 17). However, despite the expression of major histocompatibility complex I and II (MHC class I and II) and costimulatory ligands on LSCs that allow their interaction with CD4^+^ and CD8^+^ T cells (18–20), activated CTLs fail to eliminate LSCs in vivo and, rather, promote their expansion (18, 19, 21). This raises the hypothesis that the BM microenvironment may harbor immunosuppressive mechanisms that prevent the immune control of LSCs.

Tregs are essential for the maintenance of immune tolerance and represent a crucial component of the BM microenvironment during homeostasis and in leukemia (22, 23). Numbers and frequencies of Tregs in peripheral blood and BM are increased in CML patients at diagnosis (23–26). Furthermore, Tregs are especially reduced in patients who achieved a complete cytogenetic response (27). Similarly, a successful maintenance of treatment-free remission is associated with reduced numbers of Tregs (28–30). How Tregs are involved in the development of CML and immune escape of LSCs is, however, still unknown.

In this study, we analyzed the contribution of BM Tregs in the pathogenesis of CML. We show that Tregs protect LSCs from elimination by CD8^+^ CTLs and that this process can be successfully inhibited by activation of tumor necrosis factor receptor 4 (Tnfrsf4) signaling on Tregs. Overall, this study reveals TNFRSF4 as a potential target to reduce the function of Tregs and improve antileukemic immunity against LSCs.

## Results

### Thymic-derived Tregs with an activated immunophenotype accumulate in the BM of CML mice.

**Figure 1:**
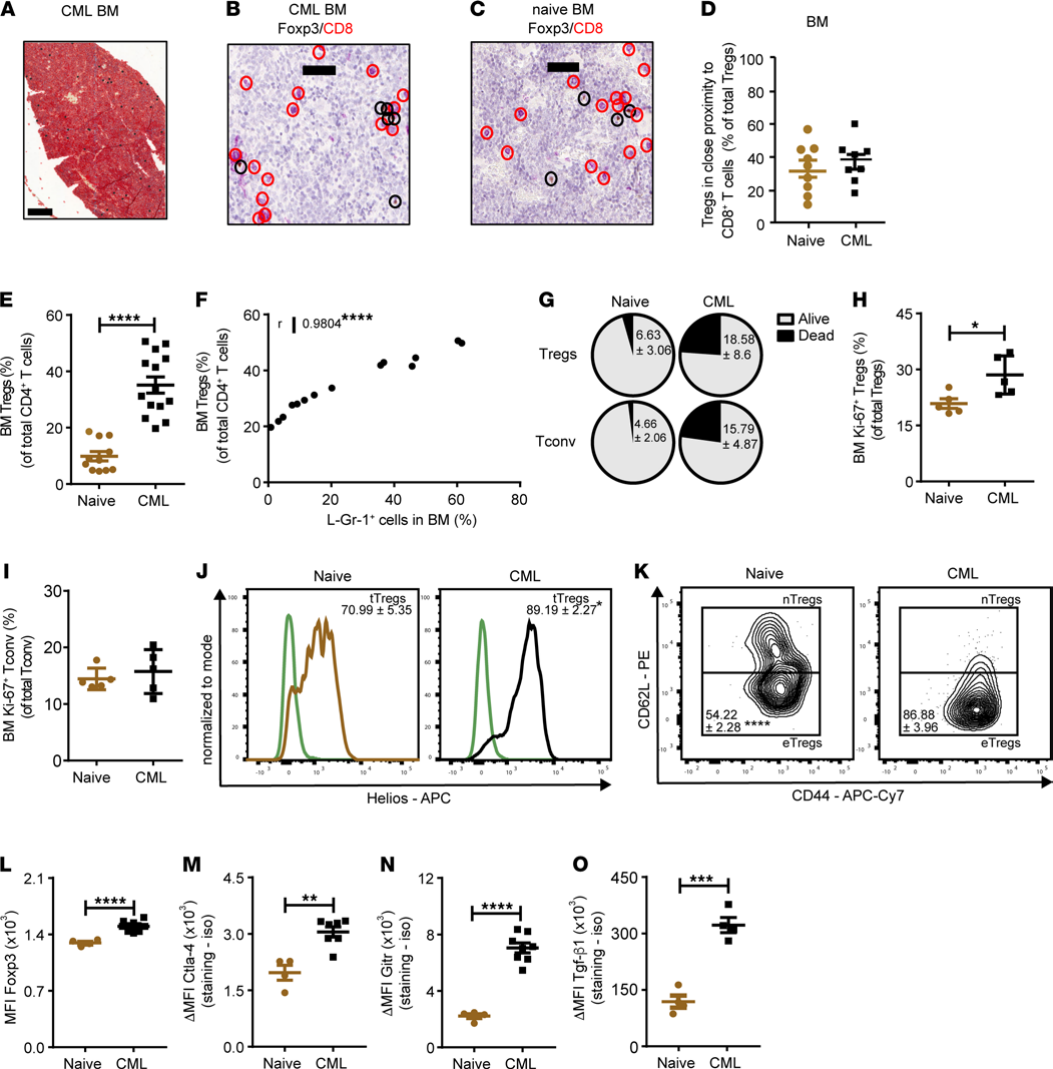
Thymic-derived effector Tregs accumulate in the BM of CML mice. (**A**) Distribution of Foxp3^+^ Tregs in CML BM (day 14). Scale bar: 200 μm; *n =* 8 mice. (**B** and **C**) Distribution of Foxp3^+^ Tregs in the BM of (**B**) CML mice (*n =* 8) and (**C**) naive mice (*n =* 9) in respect to CTLs (scale bar: 50 μm; Foxp3, brown; CD8, red). Black circles, Foxp3^+^ cells; Red circles, CTLs close to Tregs. (**D**) Frequency of Tregs located close to CTLs in the BM naive and CML mice (naive: *n =* 9 mice; CML, *n =* 8 mice). Close proximity was defined as a distance of ≤ 2 cell nuclei; *t* test. (**E**) Frequency of BM Tregs within total CD4^+^ T cell population in naive (*n =* 11) and CML (*n =* 14) *Foxp*3^DTR^ mice; *t* test. (**F**) Correlation between frequencies of Tregs (within total CD4^+^ T cells) and L-Gr-1^+^ cells in the BM of *Foxp3*^DTR^ CML mice (*n =* 14); Pearson correlation (2-sided). (**G**) Viability of Tregs and Tconv from naive and *Foxp3*^DTR^ CML mice (naive: *n =* 5 mice; CML: *n =* 9 mice); *t* test. (**H** and **I**) Proliferation of (**H**) BM Tregs and (**I**) Tconv from naive and *Foxp3*^DTR^ CML mice (naive, *n =* 5 mice; CML, *n =* 5 mice). (**J**) Representative histogram for Helios^+^ thymic-derived Tregs (tTregs) and Helios^–^ peripheral-induced Tregs (pTregs) in the BM of naive (*n =* 11) and CML *Foxp3*^DTR^ mice (*n =* 8). Pregated on CD4^+^Foxp3-GFP^+^ Tregs. Staining: beige (naive) and black (CML); isotype: green; *t* test. (**K**) Representative zebra plot for naive/resting Tregs (nTregs) and effector Tregs (eTregs) in the BM of naive (*n =* 5) and CML *Foxp3*^DTR^ mice (*n =* 5); *t* test. (**L**–**O**) MFI Foxp3 expression (GFP^+^), ΔMFI of Ctla-4, Gitr and Tgf-β1 on CD4^+^Foxp3-GFP^+^ Tregs in the BM naive (*n =* 4) and CML *Foxp3*^DTR^ mice (*n =* 4–8); *t* test. ΔMFI, staining-isotype. Data are displayed as mean ± SEM. *P < 0.05, **P < 0.01, ***P < 0.001 and ****P < 0.0001.

We first analyzed spatial distribution of BM Tregs in respect to CTLs, as well as Treg numbers and phenotype in a murine retroviral transduction/transplantation CML model (31). In the BM of CML and naive mice, Tregs were widely distributed, did not form clusters, and were preferentially localized near CTLs, as analyzed by IHC (Figure 1, A–D).

FACS analysis revealed that absolute numbers of Tregs and the frequency of Tregs among CD4^+^ T cells in the BM of CML mice were significantly increased compared with BM of naive mice (Figure 1E and Supplemental Figure 1, A and B; supplemental material available online with this article; https://doi.org/10.1172/jci.insight.151797DS1). In addition, the frequency of Tregs among CD4^+^ T cells correlated with leukemia burden (Figure 1F). The apoptosis rate of CD4^+^ T cells in the BM of CML mice was substantially higher in BM of than naive control mice. However, the apoptosis rate was similar in Tregs and CD4^+^ conventional T cells (Tconv) (annexin V^+^ cells; Tregs CML, 23.87 ± 3.67; Tconv CML, 22.81 ± 7.45) and, thus, cannot explain the increased frequency of Tregs in total CD4^+^ T cells (Figure 1, G and H). In contrast, Ki-67 staining indicated an enhanced proliferation of Tregs (Figure 1I). To determine the cellular origin of Tregs in CML, we stained for Helios and neuropilin-1, two markers that allow to discriminate Tregs that develop in the thymus (tTregs) and Tregs that arise by conversion from CD4^+^Foxp3^–^ Tconv in peripheral tissues (pTregs; ref. 32). The BM of CML mice harbored a greater proportion of tTregs (Figure 1J). In addition, Tregs in the BM of CML mice had an activated effector phenotype (eTregs) compared with controls, as indicated by an increased expression of CD44 and lack of CD62L expression on the cell surface (Figure 1K).

The accumulation of eTregs was further confirmed by assessing markers that are characteristically increased in expression during the differentiation from naive/resting Tregs (nTregs) into eTregs and that mediate their immunosuppressive function such as the transcription factor Foxp3 and the surface molecules cytotoxic T lymphocyte–associated protein 4 (Ctla-4), glucocorticoid-induced TNFR-related protein (Gitr), glycoprotein-A repetitions predominant (Garp), and transforming growth factor β1 (Tgf-β1; Figure 1, L–O). Importantly, these phenotypic changes observed in the BM of CML mice were not observed in lymphoid organs such as the spleen (Supplemental Figure 1, C–H).

### Tregs in the BM of CML mice display an activated and immunosuppressive gene expression signature.

**Figure 2:**
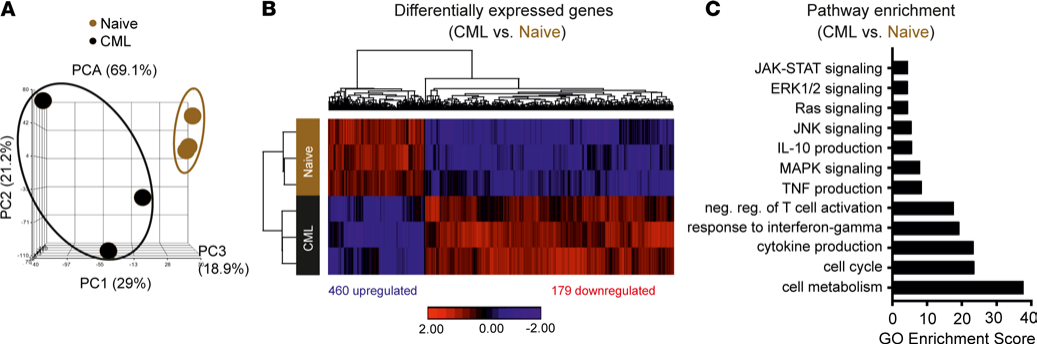
NGS RNA-Seq analysis of Tregs derived from the BM of naive and CML mice. (**A**–**C**) Principal component analysis (PCA) (**A**), heatmap of differentially expressed genes (**B**), and Gene ontology (GO) analysis (**C**) of Tregs derived from BM CML and naive *Foxp3*^DTR^ mice (*n =* 3 mice/group) upon transcriptomic analysis.

Next, we performed an RNA-Seq analysis on Tregs isolated from BM of naive and CML mice. In the principal component analysis (PCA) analysis, Tregs isolated from naive mice clustered together (Figure 2A). In contrast, Tregs derived from the BM of CML mice showed a certain degree of heterogeneity in terms of gene expression (PC2). Independent of this heterogeneity, Tregs derived from CML mice clearly separated from naive Tregs (PC1). RNA-Seq analysis identified 639 genes that were differentially expressed between the 2 groups (Figure 2B and Supplemental Table 1). In total, 460 genes were upregulated, and 179 genes were downregulated. Gene ontology (GO) analysis assigned the 639 differently expressed genes mainly into 12 different GO categories (Figure 2C). The differentially expressed genes were primarily involved in signaling pathways related to cell metabolism, cell cycle, negative regulation of T cell proliferation, and cytokine production. Overall, these results indicate a BM-specific accumulation of eTregs in CML mice.

### Depletion of Tregs eliminates LSCs and leads to long-term survival of CML mice.

**Figure 3:**
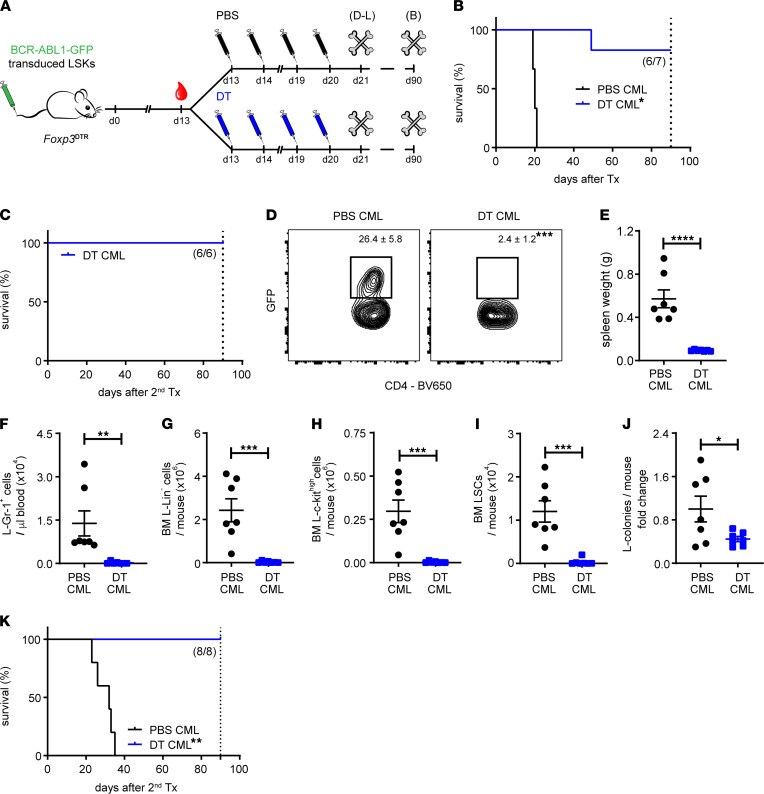
Treg depletion reduces CML LSC numbers in vivo. (**A**) Experimental setup. BCR-ABL1-GFP–transduced LSKs were injected i.v. into nonirradiated *Foxp3*^DTR^ recipients. After establishment of the disease (day 13), mice were randomized to DT or PBS treatment (days 13, 14, 19, and 20; i.p.). (**B**) Kaplan-Meier survival curves of PBS- and DT-treated CML mice (PBS, *n =* 7; DT, *n =* 7); log-rank test. (**C**) Kaplan-Meier survival curve of secondary CML mice. BM cells of surviving primary CML mice were injected i.v. into lethally irradiated secondary BL/6 recipients, and survival was monitored (*n =* 6 surviving DT CML mice). (**D**–**J**) BM CD4^+^Foxp3-GFP^+^ Tregs (**D**), spleen weight (**E**), L-Gr-1^+^ cells (**F**), and absolute numbers of L-lin^–^ cells (**G**), L–c-kit^hi^ (**H**), LSCs (**I**) in BM and colony formation capacity (**J**) per mouse was determined 21 days after CML induction (PBS: *n =* 7; DT: *n =* 8); *t* tests. (**K**) BM cells of primary CML mice (day 21) were injected i.v. into lethally irradiated secondary BL/6 recipients, and survival was monitored (PBS, *n =* 7; DT, *n =* 8); log-rank test. Data are displayed as mean ± SEM. *P < 0.05, **P < 0.01, ***P < 0.001, and ****P < 0.0001. Dotted lines represent the time point of the experiment termination at day 90.

To study the functional relevance of Tregs in CML development in vivo, we depleted Tregs in *Foxp3*^DTR^ CML mice, which express the human diphtheria toxin receptor (DTR) and enhanced GFP genes from the *Foxp3* locus, by adminis¬tration of diphtheria toxin (DT; ref. 33). *Foxp3*^DTR^ CML mice with comparable leukemia burden (37 ± 8 BCR-ABL1-GFP^+^Gr-1^+^ granulocytes; [L-Gr-1^+^ cells]/μL blood) were randomized to control treatment with vehicle or DT for Treg depletion 13 days after leukemia transplantation (Figure 3A). DT treatment resulted in the reduction of L-Gr-1^+^ cells in peripheral blood and long-term survival (Figure 3B and data not shown). In contrast, PBS-treated *Foxp3*^DTR^ CML mice all died within 30 days. No residual BCR-ABL1-GFP^+^ cells could be detected in blood, spleen, or BM of DT-treated *Foxp3*^DTR^ CML mice by FACS 90 days after transplantation (data not shown). These findings indicate that LSCs were either eliminated or effectively controlled by the depletion of Tregs. To determine residual disease with the most sensitive assay, we transplanted BM cells of surviving primary DT-treated *Foxp3*^DTR^ CML mice into lethally irradiated secondary C57BL/6J (BL/6) recipients. All secondary recipients survived up to 90 days without any signs of leukemia (Figure 3C).

To address whether LSCs are indeed affected by depletion of Tregs in our model in more detail, leukemia-bearing *Foxp3*^DTR^ mice were treated as described above with DT, and animals were sacrificed 21 days after leukemia induction. DT treatment successfully reduced/depleted Tregs in the BM of CML mice (Figure 3D). Leukemia burden — as indicated by smaller spleen size, lower numbers of L-Gr-1^+^ cells in blood, and leukemic lin^–^ and progenitor cells in the BM — was lower in DT CML mice compared with control CML mice (Figure 3, E–H). Furthermore, Treg depletion significantly reduced LSC numbers and resulted in fewer BCR-ABL1-GFP^+^ colonies formed in methylcellulose from lin^–^ BM cells (Figure 3, I and J). To functionally investigate whether leukemia-initiating cells had been eradicated, we transferred BM cells from primary CML into lethally irradiated secondary recipient mice. All secondary recipients transplanted with BM from PBS-treated primary CML developed the disease and succumbed to it with a median latency of approximately 32 days. In contrast, secondary recipients receiving BM from Treg-depleted primary CML mice survived long-term without signs of leukemia, as analyzed by FACS of peripheral blood, BM, and spleen 90 days after transplantation (Figure 3K and data not shown). Similar results on the immunophenotype of Tregs in the BM and the contribution of Tregs to leukemia development were obtained in a blast crisis CML model (Supplemental Figure 2). Overall, these results indicate that Treg depletion in a therapeutic setting contributes to the elimination of leukemia-initiating cells in vivo in mice.

### CD8^+^ CTLs selectively eliminate CML LSCs by secretion of perforin in vitro and in vivo.

**Figure 4:**
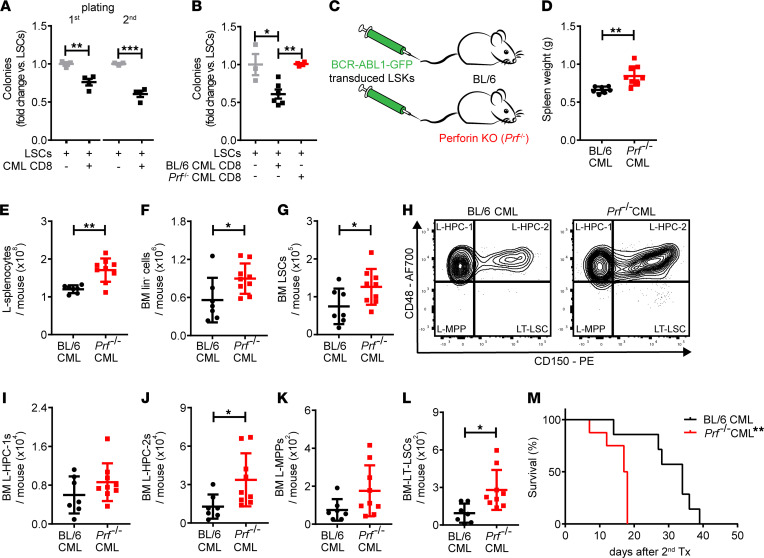
CD8^+^ CTLs from the BM of CML mice eradicate LSCs by perforin-mediated killing in vitro and in vivo. (**A**) BL/6 LSCs were cultured in the presence and absence of BM CD8^+^ CTLs from CML-bearing BL/6 overnight at a ratio of 1:1 in triplicate followed by plating in methylcellulose. Colonies were enumerated 7 days later. For secondary platings, cells isolated from primary colony assays were replated in methylcellulose in the absence of T cells; *t* test (Groups: LSCs, *n =* 3 mice; LSCs + CML CD8, *n =* 4). (**B**) BL/6 LSCs were cultured overnight in the presence and absence of CD8^+^ CTLs derived from the BM of perforin-proficient and -deficient CML mice at a ratio of 1:1 in triplicate followed by plating in methylcellulose. Colonies were enumerated 7 days later; 1-way ANOVA followed by Tukey’s multiple comparison (Groups: LSCs, *n =* 3 mice; LSCs + BL/6 CML CD8, *n =* 6; LSCs + *Prf^–/–^* CML CD8, *n =* 4). (**C**–**L**) BCR-ABL1-GFP–transduced LSKs were injected i.v. into nonirradiated BL/6 (*n =* 7) and *Prf^–/–^* (*n =* 8) recipient mice. (**D**–**G**) Spleen weight; *t* test (BL/6 CML, *n =* 7 mice; *Prf^–/–^* CML, *n =* 8 mice), absolute numbers of L-splenocytes, of L-lin^–^, and LSCs in the BM of CML mice; *t* test (BL/6 CML, *n =* 7 mice; *Prf^–/–^* CML, *n =* 8 mice) 15 days after CML induction. (**H**) Gating strategy to define LSC subpopulations; cells are pregated on lin^–^GFP^+^Sca-1^+^c-kit^+^ cells. Representative images from 1 of *n =* 7 (BL/6) and 1 of *n =* 8 *Prf^–/–^* CML mice are shown. (**I**–**L**) Absolute numbers of LSC subpopulations; *t* tests (BL/6 CML, *n =* 7 mice; *Prf^–/–^* CML, *n =* 8 mice). (**M**) BM cells from primary BL/6 (*n =* 7) and *Prf^–/–^* (*n =* 8) CML mice were injected i.v. into lethally irradiated secondary BL/6 recipients, and survival was monitored; log-rank test. Data are displayed as mean ± SEM. *P < 0.05, **P < 0.01 and ***P < 0.001.

Next, we determined whether Tregs directly regulate LSCs in CML or whether they constrain antileukemic CD8^+^ T cell immunity and thereby promote immune escape of LSCs. Therefore, we first addressed whether CD8^+^ CTLs from the BM of CML mice have the capacity to recognize and kill LSCs. We coincubated FACS-purified LSCs with CD8^+^ CTLs derived from the BM of CML-bearing mice overnight, followed by plating in methylcellulose. Coincubation of LSCs with CD8^+^ CTLs resulted in the generation of significantly fewer colonies in primary platings (Figure 4A and Supplemental Figure 3A). The negative effect on colony formation was maintained in replating experiments performed in the absence of CD8^+^ CTLs. Killing of LSCs by CD8^+^ CTLs was dependent on MHC I expression on LSCs (Supplemental Figure 3B). In contrast, CD8^+^ CTLs isolated from the BM of naive mice did not affect clonogenicity of LSCs (data not shown). Overall, these data suggest that BM CD8^+^ CTLs have the capacity to kill LSCs in vitro.

The accepted hallmark of a fully active CD8^+^ CTL remains its perforin-killing machinery, even though they exhibit both Fas ligand–based (FasL-based) and perforin-based lytic activities (34). To investigate if BM CD8^+^ CTLs reduce LSCs through perforin-mediated killing in CML, we coincubated LSCs in the presence of CD8^+^ CTLs derived from the BM of perforin-proficient and -deficient CML mice. In contrast to coincubation with perforin-proficient CD8^+^ CTLs, coincubation with perforin-deficient CML CD8^+^ CTLs did not reduce colony formation (Figure 4B). Similarly, the exposure of LSCs to the granzyme B inhibitor I prior to coculture with CD8^+^ CTLs protected LSCs from MHC I–dependent CD8^+^ CTL–mediated killing in vitro (Supplemental Figure 3, B and C). Importantly, the clonogenic potential of normal lin^–^c-kit^+^sca-1^+^ (LSK) hematopoietic stem/progenitor cells (LSKs) derived the BM of naive BL/6 mice was not affected by coincubation of LSKs with CD8^+^ CTLs from CML mice (Supplemental Figure 3D).

Lastly, we induced CML in BL/6 and perforin-deficient mice (BL/6 CML and *Prf^–/–^* CML, respectively; Figure 4C). Fifteen days after leukemia induction, mice were sacrificed, and BM and spleens were analyzed. *Prf^–/–^* CML mice had an increased leukemia burden, as indicated by bigger spleen size, higher numbers of BCR-ABL1-GFP^+^ leukemia splenocytes (L-splenocytes), and of BCR-ABL1-GFP^+^lineage^–^ (L-lin^–^) cells in the BM compared with BL/6 CML mice (Figure 4, D–F). Similarly, we found a strong increase in LSC numbers in the BM of *Prf^–/–^* CML mice (Figure 4G). LSCs can be further subdivided into long-term LSCs (LT-LSCs), leukemia multipotent progenitors (L-MPPs), and leukemia progenitor cells (L-HPC-1s and L-HPC-2s) using the markers CD150 and CD48 (18, 35). Phenotypic LSC subset analysis revealed that the increase of LSCs in *Prf^–/–^* CML mice was, in great part, mediated by a significant accumulation of L-HPC-2 cells and, more importantly, of disease-initiating and -maintaining LT-LSCs (Figure 4, H–L). Animals transplanted with BM from *Prf^–/–^* CML mice in secondary transplantation experiments succumbed to the disease significantly faster than mice transplanted with BM from BL/6 control CML mice (Figure 4M). Overall, these data suggest that CD8^+^ CTLs can recognize and eliminate CML LSCs.

### Tregs protect LSCs from CD8^+^ CTL–mediated killing in vitro and in vivo.

**Figure 5:**
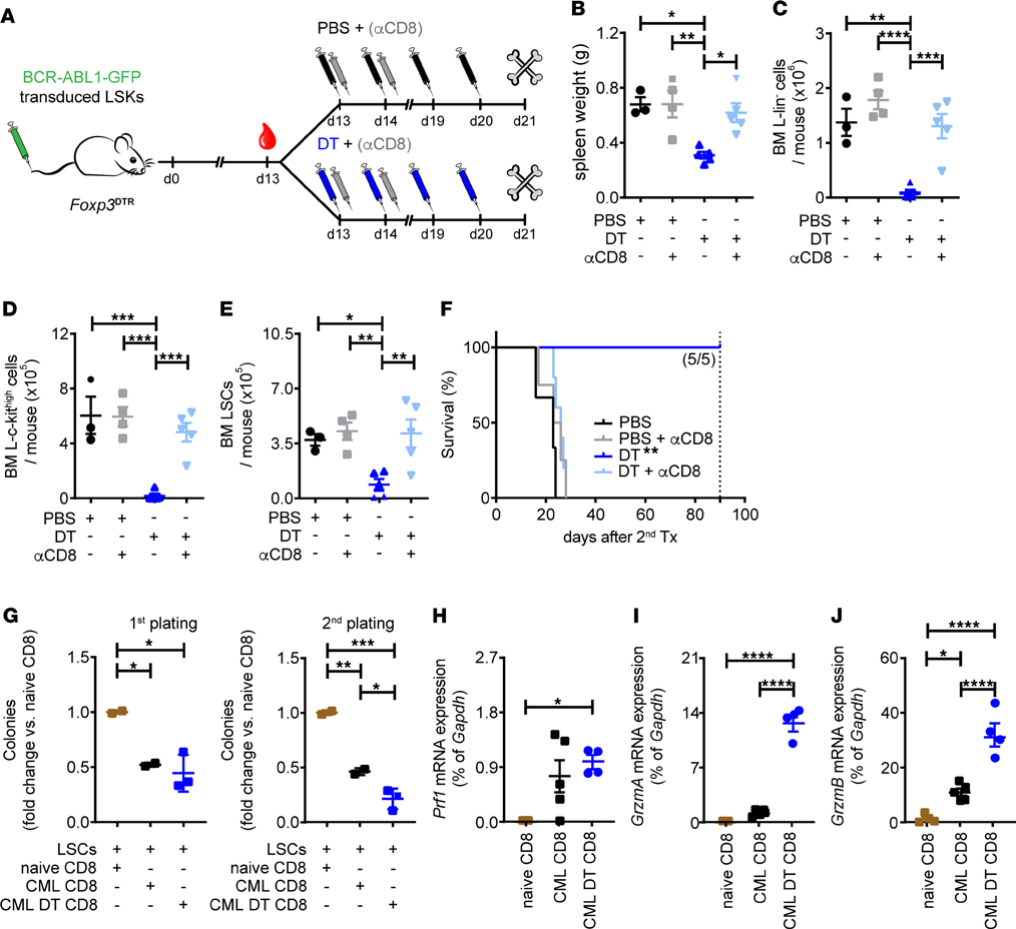
BM Tregs in CML protect LSCs from CD8^+^ T cell–mediated eradication in vivo. (**A**) Experimental setup. BCR-ABL1-GFP–transduced LSKs were injected i.v. into nonirradiated *Foxp3*^DTR^ recipients. At day 13, mice were randomized to PBS/DT and αCD8 mAb treatment (DT i.p. at days 13, 14, 19, and 20; αCD8 mAb at days 13 and 15 i.p.). (**B**–**E**) Spleen weight, absolute numbers of L-lin^–^ cells, L–c-kit^hi^ cells, and LSCs in the BM of CML mice of all treatment groups 21 days after CML induction; 1-way ANOVAs followed by Tukey’s multiple comparison test. PBS, *n =* 3 mice; PBS + αCD8, *n =* 4 mice; DT, *n =* 5 mice; and DT + αCD8, *n =* 5 mice. (**F**) BM cells of primary CML mice (day 21) were injected i.v. into lethally irradiated secondary BL/6 recipients, and survival was monitored; log-rank test. PBS, *n =* 3 mice; PBS + αCD8, *n =* 4 mice; DT, *n =* 5 mice; and DT + αCD8, *n =* 5 mice. (**G**) LSCs were preincubated with CD8^+^ CTLs from naive or CML-bearing mice treated with PBS or DT overnight in a 1:1 ratio, followed by plating in methylcellulose. Myeloid CFU and replating capacity in vitro (*n =* 3 mice/group); 1-way ANOVA followed by Tukey’s post hoc test. (**H**–**J**) Perforin (*Prf1*), Granzyme A (*GrzmA*), and Granzyme B (*GrzmB*) mRNA expression levels in BM CD8^+^ CTLs measured by qPCR. Data are normalized to *Gapdh* (naive, *n =* 4 mice; CML, *n =* 5 mice; CML DT, *n =* 4 mice); 1-way ANOVA followed by Tukey’s post hoc test. Data are displayed as mean ± SEM. *P < 0.05, **P < 0.01, ***P < 0.001, and ****P < 0.0001. Dotted line represents the time point experiment termination day 90.

To prove that Tregs in the BM constrain antileukemic CD8^+^ T cell immunity in CML, *Foxp3*^DTR^ CML mice were treated 13 days after CML induction with either PBS, DT, or a depleting αCD8 mAb (PBS/αCD8) alone or in combination (DT/αCD8; Figure 5A). Depletion of CD8^+^ T cells alone did not affect leukemia load in the spleen (Figure 5B). Similarly, numbers of L-lin^–^ cells, L–c-kit^hi^, cells and LSCs in the BM were comparable to PBS-treated control CML mice after CD8^+^ T cell depletion (Figure 5, C–E). In line with the findings depicted in Figure 2, Treg depletion by DT administration considerably reduced leukemia load and LSC numbers in the BM (Figure 5, C–E, and Supplemental Figure 4A). In contrast, DT/αCD8 treatment restored leukemia burden and LSC numbers in BM to levels comparable with PBS and αCD8/PBS-treated CML mice. These findings were confirmed functionally by secondary transplantation experiments (Figure 5F).

Similarly, coculture experiments revealed that CD8^+^ CTLs fail to eliminate LSCs in vitro in the presence of Tregs derived from the BM of CML but not from naive mice (Supplemental Figure 3E). In addition, coincubation with CML Tregs alone did not alter the clonogenic potential of LSCs in vitro.

Lastly, we investigated whether BM CD8^+^ CTLs from DT-treated CML mice are more potent in eliminating LSCs in vitro. Thus, we cocultured LSCs with BM CD8^+^ CTLs from naive mice and PBS- or DT-treated CML mice overnight, followed by plating in methylcellulose. Coincubation of CD8^+^ CTLs from DT-treated CML mice even further reduced the clonogenic potential of LSCs compared with CD8^+^ CTLs from PBS-treated CML mice (Figure 5G). In addition, the expression of genes related to the capacity of CD8^+^ CTLs to lyse and kill LSCs such as *GrzmA* and *GrzmB* were significantly increased in CD8^+^ CTLs derived from DT-treated CML mice compared with CD8^+^ CTLs from PBS-treated CML mice (Figure 5, H–J). These data suggest that Tregs in the BM indirectly promote immune escape of LSCs through modulation of CD8^+^ CTL activity.

### Tregs in CML are activated by antigens presented on MHC class II–expressing LSCs.

The expression of cognate antigens triggers the differentiation of tTregs (36–38). To determine whether leukemia cells, and especially LT-LSCs, have the capacity to interact with and activate Tregs via MHC class II/TCR interaction, we assessed the expression of MHC class II on LSC subsets and more differentiated leukemia and progenitor cells by FACS. MHC class II was strongly expressed on LSC subsets, including LT-LSCs. In contrast, leukemia progenitor and fully differentiated L-Gr-1^+^ cells had reduced levels of MHC class II expressed on the cell surface (Supplemental Figure 4B). These results indicate that especially LSCs possess the capacity to interact and activate tTregs in our CML mouse model.

**Figure 6:**
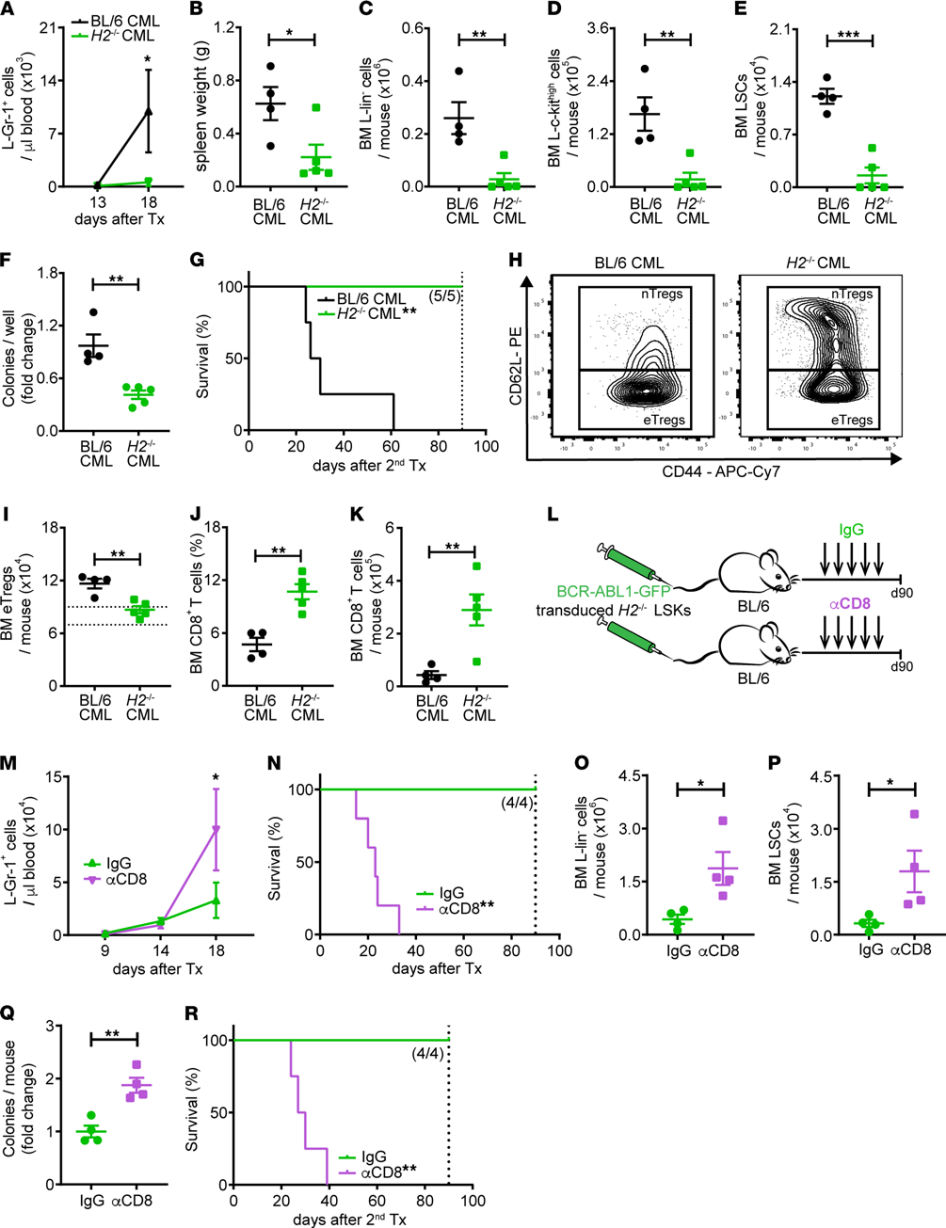
Tregs in the BM of CML mice are activated through interaction with MHC class II on leukemia cells resulting in immune escape of LSCs. (**A**) L-Gr-1^+^ cells in blood from BL/6 and MHC class II–deficient (*H2^–/^)^–^* CML mice (*n =* 4–5 mice/group); 2-way ANOVA followed by Sidak’s multiple comparison test. (**B**–**E**) Spleen size, numbers of (**C**) L-lin^–^, (**D**) L–c-kit^hi^ cells and (**E**) LSCs in BM and (**F**) Colony formation capacity per mouse of BL/6 and *H2^–/–^* CML mice (18 day); Student’s *t* test. (**G**) BM cells of primary CML mice (day 18) were injected i.v. into lethally irradiated secondary BL/6 recipients, and survival was monitored; log-rank test. (**H**) Gating strategy to identify nTregs and eTregs; pregated on CD4^+^ Foxp3^+^ Tregs. (**I**) Numbers of eTregs within CD4^+^ T cell population in BL/6 and *H2^–/–^* CML. Dotted lines: range of eTregs observed in naive mice (*n =* 5); Student’s *t* test. (**J** and **K**) Frequencies and numbers of CD8^+^ T cells in BL/6 and *H2^–/–^* CML mice (day 18); *t* test. (**L**) BL/6 mice were treated i.p. with an αCD8 mAb (75 μg/injection) or control IgG at days –2, –1, 4, 9, and 14 (Groups: IgG, *n =* 4; αCD8, *n =* 5 mice/group). (**M** and **N**) Number of L-Gr-1^+^ cells in the blood and Kaplan-Meier survival graph of IgG- or αCD8-treated *H2^–/–^* CML mice (Groups: IgG, *n =* 4; αCD8, *n =* 5 mice/group); 2-way ANOVA followed by Sidak’s multiple comparison test and log-rank test. (**O**–**Q**)Number of L-lin^–^ cells and LSCs in the BM and colony formation capacity per mouse of IgG-treated and αCD8-treated *H2^–/–^* CML mice (day 16; *n =* 4 mice/group); *t* test. (**R**) Kaplan-Meier survival graph from mice receiving BM cells of primary CML mice (day 16; *n =* 4 mice/group); log-rank test. Data are displayed as mean ± SEM. *P < 0.05, **P < 0.01, and ***P < 0.001. Dotted lines indicate time point experiment termination day 90.

To address whether a lack of MHC class II on LSCs affects Treg activation and, consequently, disease development in our CML model, we transplanted MHC class II–proficient (*H2*) and –deficient BCR-ABL1-GFP–transduced LSKs into nonirradiated *Foxp3*^DTR^ mice. Even though *H2^–/–^* and BL/6 LSCs did not differ in their potential to form colonies in primary and secondary replating experiments in vitro (Supplemental Figure 4C), leukemia developed significantly slower in *H2^–/–^* CML mice compared with BL/6 CML mice, as indicated by considerably lower levels of L-Gr-1^+^ cells in peripheral blood (Figure 6A). Eighteen days after leukemia induction, CML mice of both groups were sacrificed, and spleen and BM were analyzed. Spleen size was significantly smaller in *H2^–/–^* CML mice compared with controls, indicating a lower leukemia burden in these mice (Figure 6B). Phenotypic analysis of lin^–^ BM cells by FACS further revealed significantly fewer L-lin^–^ and L–c-kit^hi^ cells and a 7-fold reduction of LSCs in *H2^–/–^* CML mice (Figure 6, C–E), a finding that was functionally confirmed by colony assays of lin^–^ BM cells in vitro (Figure 6F). To verify that the decrease in LSCs detected by FACS analysis and in colony forming assays in vitro represents a reduction in cells that can induce leukemia in vivo, we secondarily transplanted BM cells from primary BL/6 and *H2^–/–^* CML mice into lethally irradiated secondary BL/6 recipient mice. Mice that received BM from BL/6 leukemia mice developed a more severe course of the disease and died with a median latency of 29 days. In contrast, mice that were transplanted with BM cells from primary *H2^–/–^* CML mice survived long-term without any signs of leukemia (Figure 6G).

Analysis of the activation state of Tregs in the BM of primary BL/6 and *H2^–/–^* CML mice revealed fewer eTregs in the BM of CML mice in the absence of MHC class II expression on LSCs. Importantly, the frequency of eTregs in these mice was comparable with the eTreg frequency in the BM of naive mice (Figure 6, H and I). The reduced activation of Tregs in *H2^–/–^* CML was complemented by a significant increase in the frequency and absolute numbers of CD8^+^ T cells (Figure 6, J and K).

### CD8^+^ CTL depletion renders H2^–/–^ CML mice susceptible to disease development.

Based on these results, we speculated that blockade of CD8^+^ CTL activity or depletion of CD8^+^ T cells would render *H2^–/–^* CML mice susceptible to CML development. To test this hypothesis, we depleted CD8^+^ CTLs in *H2^–/–^* CML mice by repetitive treatment with an αCD8 mAb (Figure 6L). While IgG-treated control *H2^–/–^* CML mice were protected from CML development and survived long-term, CD8^+^ CTL depletion completely restored the competence of *H2^–/–^* CML mice to develop leukemia and resulted in death of the mice approximately 20–30 days after transplantation (Figure 6, M and N). To further determine the effect of CD8 blockade on LSCs in *H2^–/–^* CML, αCD8 mAb– or control IgG–treated *H2^–/–^* CML mice were sacrificed 16 days after CML induction, and BM was analyzed. CD8^+^ CTL depletion significantly increased leukemia burden, as demonstrated by an elevated number of L-lin^–^ cells and LSCs, assessed phenotypically by FACS and functionally by both colony formation assays and secondary transplantation of BM into secondary recipients (Figure 6, O–R). These results indicate that Tregs are activated by antigens presented on MHC class II–expressing leukemia cells and LSCs.

### Stimulation of Tnfrsf4 signaling reduces the capacity of Tregs to protect LSCs from CD8^+^ CTL–mediated killing in CML.

**Figure 7:**
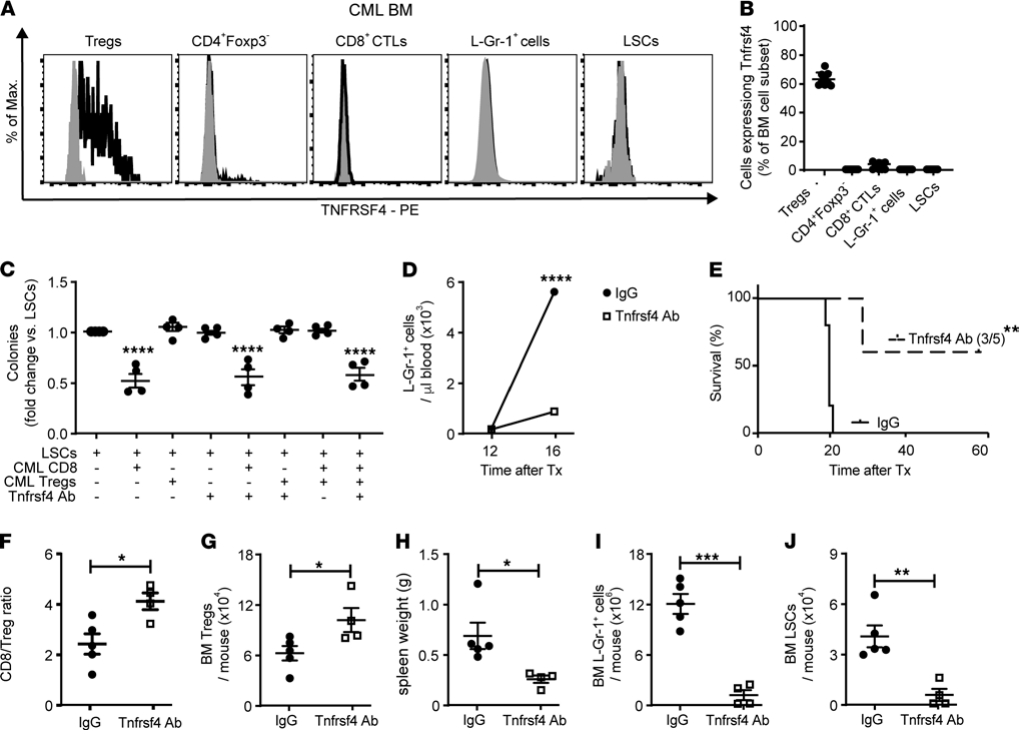
Stimulation of Tnfrsf4-signaling reduces the capacity of Tregs to protect LSCs from CD8^+^ CTL–mediated killing. (**A**) Representative FACS plots for the expression of Tnfrsf4 on CD4^+^Foxp3-GFP^+^ T cells (Tregs), CD4^+^Foxp3^–^ T cells, CD8^+^ CTLs, L-Gr-1^+^ cells, and LSCs in the BM of *Foxp3*^DTR^ CML mice. One representative out of 9 plots is depicted. Staining, black; isotype control, gray. (**B**) Frequency of CD4^+^Foxp3-GFP^+^ T cells, CD4^+^Foxp3^–^ T cells, CD8^+^ CTLs, L-Gr-1^+^ cells, and LSCs in the BM of CML mice expressing Tnfrsf4 (*n =* 9 mice/cell subset). (**C**) LSCs from the BM of *Foxp3*^DTR^ CML mice were cultured with BM CD8^+^ T cells and/or BM Tregs of the same mice pretreated for 2 hours with a Tnfrsf4 antibody (clone OX-86, 30 μg/mL) or respective control antibody at a ratio of 1:1:1 in triplicate followed by plating in methylcellulose. Colonies were enumerated 7 days later; 1-way ANOVA followed by Tukey’s post hoc test. (**D** and **E**) BL/6 CML mice were randomized to control IgG or anti-Tnfrsf4 antibody treatment (OX-86, 200 μg/mouse, i.p, for 6 times every second day, starting at day 12), and leukemia development and survival was monitored. (**D**) Number of L-Gr-1^+^ cells in the blood of IgG-treated and Tnfrsf4 antibody–treated BL/6 CML mice; 2-way ANOVA followed by Sidak’s multiple comparison test (*n =* 5 mice/group). (**E**) Kaplan-Meier survival curves of control IgG– and TNFRSF4 Ab–treated CML mice (IgG, *n =* 5; Tnfrsf4, *n =* 5); log-rank test. (**F**–**J**) CD8/Treg ratio in the BM, Treg numbers in the BM, spleen weight, numbers of L-Gr-1^+^ cells, and LSCs in the BM of control IgG– and TNFRSF4 Ab–treated CML mice (IgG, *n =* 5; Tnfrsf4, *n =* 4) 18 days after CML transplantation. *t* test. Data are displayed as mean ± SEM. *P < 0.05, **P < 0.01, ***P < 0.001, and ****P < 0.0001. One representative out of 2 independent experiments is shown.

Next, we determined whether immune-related surface receptors that were upregulated in CML BM could be used to selectively target Tregs. Among the most upregulated genes, our RNA-Seq analysis identified 5 immune-related surface receptors (*Tnfrsf1b*, *Tigit*, *Tnrsf4*, *Tnfrsf8*, and *Tnfrsf9*; Supplemental Table 1). Because *Tnfrsf1b* and *Tnfrsf9* have a reported role in the regulation of normal hematopoietic stem cells (39) and myeloid differentiation of early hematopoietic progenitor cells (40, 41), we focused our subsequent analysis on *Tigit*, *Tnrsf4*, and *Tnfrsf8*. FACS analysis revealed that — besides CD4^+^Foxp3^+^ Tregs, also a fraction of CD8^+^ CTLs — CD4^+^Foxp3^–^ T cells, L-Gr-1^+^ cells, and LSCs express Tigit in the BM of CML mice (Supplemental Figure 4, D and E). In contrast, Tnfrsf8 (alias CD30) was absent on the protein level on all cell populations analyzed, including CD4^+^Foxp3^+^ Tregs (data not shown). Tnfrsf4 could not be detected on the surface of CD8^+^ T cells, L-Gr-1^+^ cells, or LSCs, while a substantial fraction of CD4^+^Foxp3^+^ Tregs and a minor fraction of CD4^+^Foxp3^–^ T cells expressed Tnfrsf4 in the BM of CML mice (Figure 7, A and B). These data suggest that Tnfrsf4 may serve as a target to selectively eliminate/inactivate Tregs in CML without directly affecting CD8^+^ CTL–mediated immunity and leukemia cells. To proof this concept, we cocultured LSCs and CD8^+^ CTLs from CML BM in the presence and absence of CML Tregs and an agonistic Tnfrsf4 antibody followed by plating in methylcellulose. The agonistic Tnfrsf4 antibody OX86 has been shown to mediate Tnfrsf4 forward signaling on Tregs, leading to their functional inactivation in vitro and in vivo (42–44), and it has also been demonstrated to deplete Tnfrsf4-expressing Tregs in other solid tumor models (45). CD8^+^ CTLs reduced colony formation of LSCs independently of the presence of the antibody. In contrast, addition of the antibody to the coculture of LSCs, Tregs, and CD8^+^ CTLs reduced colony formation of LSCs to levels comparable with cocultures of LSCs and CD8^+^ CTLs. Colony formation of LSCs was not affected by addition of the antibody into the monoculture (Figure 7C).

To demonstrate the in vivo relevance of our findings, BL/6 CML mice were treated with either control IgG or an agonistic Tnfrsf4 antibody starting at day 12 day after CML induction, and disease development was monitored. Tnfrsf4 antibody treatment reduced L-Gr-1^+^ cells in the peripheral blood and significantly prolonged survival of CML mice with 60% of mice surviving long-term (Figure 7, D and E). Mechanistically, Tnfrsf4 antibody treatment significantly increased the CD8/Treg ratio in BM without depleting/reducing Treg numbers (Figure 7, F and G), which resulted in reduced leukemia and reduced numbers of BM LSCs (Figure 7, H–J). Overall, these data indicate that triggering of TNFRS4 signaling on Tregs promotes antileukemic immunity and promotes elimination of CML LSCs by CD8^+^ CTLs.

### Tregs protect primary human CD34^+^CD38^–^ CML stem/progenitor cells from CD8^+^ CTL–mediated killing in vitro.

**Figure 8:**
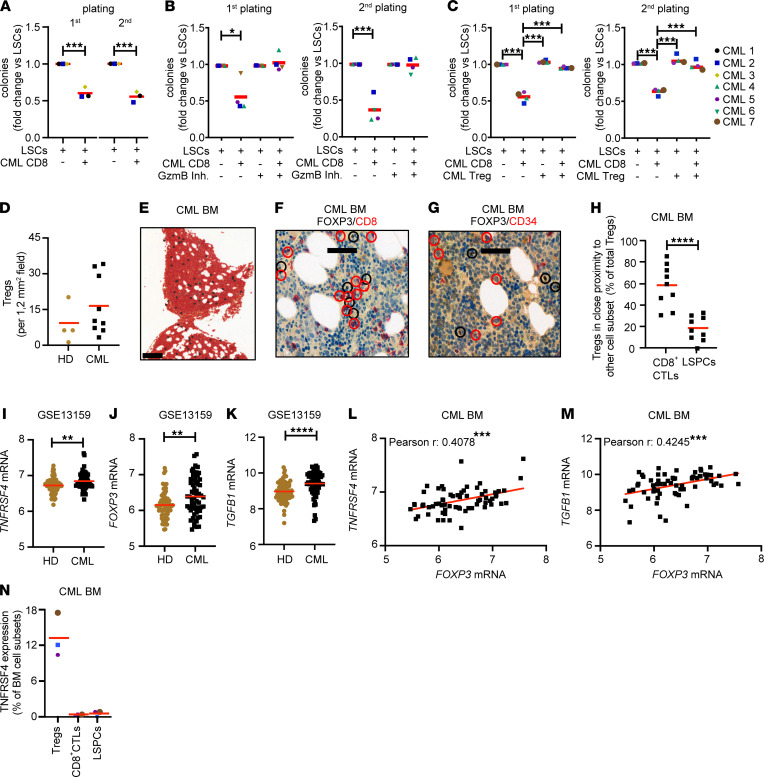
Tregs protect human CD34^+^CD38^–^ CML stem/progenitor cells from elimination by CD8^+^ CTLs. (**A**) Colony forming and replating capacity of CD34^+^CD38^–^ LSCs (CML 1–3) cultured overnight in the presence and absence of CD8^+^ CTLs of the same CML patients at a ratio of 1:1; *t* test. (**B**) Colony forming and replating capacity of LSCs (CML 4–7) pretreated with the granzyme B inhibitor I (100 μM) and cultured in the presence and absence of CD8^+^ CTLs at a ratio of 1:1 overnight; *t* test. (**C**) Colony forming and replating capacity of LSCs (CML 4–6) cultured in the presence and absence of CD8^+^ CTLs and/or CD25^+^CD127^lo^ CD4^+^ Tregs at a ratio of 1:1:1. One-way ANOVA followed by Tukey’s post hoc test. (**D**) Numbers of BM Tregs in CML patients and healthy donors (CML, *n =* 10; healthy donors [HD], *n =* 4). Tregs per 1.2 mm^2^ field were determined; *t* tests. (**E**) Distribution of BM FOXP3^+^ Tregs (scale bar: 200 μm; *n =* 10 CML patients). (**F** and **G**) Spatial localization of BM FOXP3^+^ Tregs in respect to (**F**) CD8^+^ CTLs and (**G**) CD34^+^ CML stem/progenitor cells (LSPCs) (scale bar: 50 μm; *n =* 10 CML patients; FOXP3, brown; CD8^+^ CTLs and LSPCs, red). FOXP3^+^ cells, black; CD8^+^ CTLs and LSPCs, red circles. (**H**) Frequency of BM Tregs located near CD8^+^ CTLs and LSPCs (*n =* 10 CML patients). Close proximity was defined as a distance of ≤ 2 cell nuclei; *t* tests. (**I**–**K**) *TNFRSF4*, *FOXP3*, and *TGFB1* mRNA expression in CML patients and HD (*n =* 73; CML: *n =* 76; GSE13159); *t* test. (**L** and **M**) Correlation of *FOXP3* with (**L**) *TNFRSF4* and (**M**) *TGFB1* mRNA expression in BM of CML patients (*n =* 76; GSE13159); Spearman correlations. (**N**) Frequency of CD25^+^CD127^lo^ CD4^+^ Tregs, CD8^+^ CTLs, and LSPCs expressing TNFRSF4 on the cell surface in CML patients (CML 2, 5, 7) analyzed by FACS. Data are displayed as mean. *P < 0.05, **P < 0.01, ***P < 0.001 and ****P < 0.0001.

To validate the significance of our findings for human CML, we addressed whether CD8^+^ CTLs can kill CD34^+^CD38^–^ CML stem/progenitor cells derived from newly diagnosed chronic phase CML patients and whether this effect can be reverted in the presence of Tregs (Supplemental Table 2). Therefore, we first coincubated FACS-purified CML stem/progenitor cells overnight with FACS-purified CD8^+^ CTLs derived from the same patients at an effector/target ratio of 1:1, followed by plating in methylcellulose. Like our results obtained with mice, coincubation with CD8^+^ CTLs reduced the clonogenic potential of primary CML stem/progenitor cells in a granzyme-dependent manner (Figure 8, A and B, and Supplemental Table 2). Importantly, addition of Tregs to the culture of CD8^+^ CTLs and CML stem/progenitor cells prevented elimination of CML stem/progenitor cells by CD8^+^ CTLs (Figure 8C). In contrast, coculture of LSCs with Tregs did not affect their clonogenic potential. Overall, these data indicate that Tregs in the BM protect LSCs from elimination by CD8^+^ CTLs in CML.

### Tregs are increased in BM of newly diagnosed CML patients and are located close to CD8^+^ CTLs.

In line with previous findings (23), analysis of BM sections from a limited number of CML patients and healthy donors by IHC demonstrated that Treg numbers tend to be increased in CML BM (Figure 8D). Tregs were widely distributed in the BM parenchyma in CML and healthy conditions (Figure 8E and Supplemental Figure 5A). While a comparable frequency of about 30% Tregs were found close to normal CD34^+^ stem/progenitor cells and CD8^+^ CTLs in the healthy donor BM (Supplemental Figure 5, B–D), the majority of Tregs in BM of CML patients were close to CD8^+^ CTLs (58.44% ± 6.53%) but not CD34^+^ CML stem/progenitor cells (18.33% ± 3.87%) (Figure 8, F–H)

### TNFRSF4 mRNA expression is increased in the BM of CML patients.

To demonstrate a role for TNFRSF4 in CML, we analyzed mRNA expression of *TNFRSF4* and Treg-associated genes such as *FOXP3* and *TGFB1* in the BM of 66 newly diagnosed chronic phase CML patients and 73 healthy controls using a publicly available microarray data set (GSE13159; https://www.ncbi.nlm.nih.gov/geo/query/acc.cgi?acc=GSE13159). We found the expression of *TNFRSF4*, *FOXP3*, and *TGFB1* mRNA significantly increased in BM samples from CML patients compared with controls (Figure 8, I–K). Importantly, the expression of *FOXP3* mRNA strongly correlated with *TNFRSF4* and *TGFB1* in the BM of CML patients but not in healthy donor control BM (Figure 8, L and M, and Supplemental Figure 5, E and F). FACS analysis of the BM from a limited number of newly diagnosed CML patients revealed that a significant fraction of CD4^+^CD127^lo^CD25^+^ BM Tregs express the TNFRSF4 on the surface, whereas TNFRSF4 was absent on CD8^+^ CTLs and CML stem/progenitor cells (Figure 8N and Supplemental Figure 5G).

## Discussion

Leukemia can only be eradicated long-term by targeting disease-initiating and -maintaining LSCs (5, 46). Despite the clinical success of TKIs in the treatment of CML patients, quiescent, TKI-resistant LSCs remain in the BM in a majority of patients and can cause relapse of the disease after drug discontinuation or through the acquisition of mutations (5). For these patients, immunotherapy might be a potential therapeutic option. However, LSCs also seem resistant to elimination by activated CD8^+^ CTLs in vivo, and various immune effector mechanisms contribute to the expansion of LSCs rather than to their elimination (18, 19, 47, 48). Why LSCs are selectively resistant against elimination by CD8^+^ CTLs is still unknown.

In the present study, we describe Tregs in the BM as an important mediator of immune escape of LSCs in CML. During homeostasis, Tregs are enriched in the BM and are thought to provide an immune-privileged niche, protecting hematopoietic stem and progenitor cells (HSPCs) from immune destruction (22). In addition, Camacho et al. recently demonstrated that BM Tregs regulate hematopoiesis indirectly through modulation of stromal cell function (49). In CML, numbers and frequencies of Tregs in peripheral blood and BM are increased in patients at diagnosis and correlate with a poor prognosis (Sokal score; refs. 23–26). In addition, Treg numbers further increase in accelerated phase and blast-crisis CML patients compared with chronic phase CML patients (25). In line with these findings, we document that Tregs are increased in CML BM in frequency and absolute numbers in a murine CML model. Depletion of Tregs in a therapeutic setting through short-term administration of DT in *Foxp3*^DTR^ mice resulted in activation of CD8^+^ CTLs, elimination of LSCs, and long-term survival. In addition, coculture of Tregs from the BM of CML mice but not from BM of naive mice with LSCs and CD8^+^ CTLs prevented the killing of LSCs in vitro. The results obtained in mice were confirmed in comparable experiments using Treg, CML stem/progenitor cells and CD8^+^ CTLs from newly diagnosed CML patients and suggest a similar role of Tregs in the protection of LSCs from CD8^+^ CTLs–mediated killing in humans.

Our study describes the distribution and the spatial localization of Tregs in the BM during homeostasis and in CML. Tregs were widely distributed throughout the BM parenchyma in mice and humans. In human CML, the majority of Tregs were localized close to CD8^+^ CTLs and not close to CD34^+^ CML stem/progenitor cells. Similarly, a big proportion of Tregs was in close proximity to CD8^+^ CTLs in the BM of CML mice. Overall, our findings suggest that Tregs in CML BM preferentially interact with CD8^+^ CTLs and regulate their function instead of interacting directly with CML stem/progenitor cells — findings that are supported by functional data generated in this study.

Tregs in CML BM were activated, thymic-derived, and overexpressed receptors such as Ctla-4, Gitr, Garp, and Tgf-β1 on the surface that have been previously reported to mediate their activity and immunosuppressive function in various cancer entities (50). Given that TCR stimulation is required for activation and acquisition of suppressive function in Tregs (36–38), the activated profile of BM Tregs in our study suggests that antigen stimulation may play an important role in the activation and accumulation of Tregs in CML BM. In line with this hypothesis, we found that MHC class II expression on LSCs promoted the activation and accumulation of eTregs in the BM, resulting in immune escape of LSCs from CD8^+^ CTL–mediated immunity in CML. In CML patients, leukemia-antigen specificity of CD4^+^ T cells has been documented in several independent studies (17, 51–53). However, whether CML antigen–specific Tregs are part of this CD4^+^ T cell population is still unclear. In general, the evidence for functional tumor antigen–specific Tregs in cancer is very weak due to the lack of adequate MHC class II tetramers, and antigen-specific Tregs have only been documented in a few solid tumors and in B acute lymphoblastic leukemia (54–58).

CML has a lower mutational burden compared with most solid tumors and, therefore, has a lower number of neoantigens that can be recognized by specific CD8^+^ CTLs (59), suggesting that LSCs in myeloid leukemia may have a low degree of immunogenicity. Similarly, the frequency of CML-specific CD8^+^ CTLs at diagnosis in humans is rather low (14, 60). Here, we document for the first time to our knowledge that CML LSCs can be recognized and killed by antigen-specific BM CD8^+^ CTLs through perforin/granzyme-mediated lysis, even though only a minority of the BM CD8^+^ CTLs are leukemia specific.

In contrast, CD8^+^ CTLs may also contribute to the expansion of LSCs, as documented in earlier studies (18, 19, 47, 48). This discrepancy may be explained by differences in the activation status of the specific T cells, the effector/target ratio, and, as shown in our present study, the presence of Tregs (18, 19, 47, 48). For example, the transfer of a large numbers of activated T cell receptor transgenic T cells leads to a IFN-γ–dependent expansion of LSCs, whereas the physiological activation of few CML-specific CD8^+^ T cells leads to the elimination of LSCs (19).

Tregs are an important regulator of homeostasis in the BM and provide an immune privilege niche for HSCs (49, 61). Due to the crucial role of Tregs in the regulation of the BM microenvironment, unselective targeting of Tregs would seriously affect normal hematopoiesis. To identify surface markers that can be selectively targeted on Tregs in the BM of CML mice, we performed an RNA-Seq analysis of BM Tregs from CML and naive mice. In line with our phenotypic observations, Tregs in the BM of CML mice had an enhanced expression of genes related to Treg differentiation and function, cell cycle, inflammation, and immunosuppression. We identified the TNFRSF4 as a cell surface receptor that was highly overexpressed on CML Tregs at mRNA level. Activation of TNFRSF4 forward signaling by the agonistic antibody OX86 did not deplete Tregs but reduced the immunosuppressive function of Tregs and, thereby, inhibited the capacity of BM Tregs to protect LSCs from elimination by CD8^+^ CTLs. TNFRSF4 agonists are currently being investigated alone or in combination with other immunotherapies for the treatment of various tumor entities (50). Consequently, the efficacy of an agonistic TNFRSF4 antibody to modulate T cell immunity and to eliminate LSCs in CML patients could be directly addressed in patients who did not obtain a deep molecular remission, as well as in patients who relapsed after discontinuation of TKI therapy. In summary, our study identifies Tregs as central regulators of immune escape of LSCs and identifies TNFRSF4 as a potential target to modulate the Tregs and promote antileukemic immunity in CML.

## Methods

### Antibodies for flow cytometry

Mouse antibodies were used. αLy-6A/E-PerCP-Cy5.5 (Sca-1, clone D7; 1:600, catalog 108123, RRID:AB_893619), αCD117-APC-Cy7 (c-kit, clone 2B8, 1:300, catalog 105838, RRID:AB_2616739), αLy6G/C- PE (clone RB6-8C5, 1:400, catalog 108408, RRID:AB_313373), αCD19-APC-Cy7 (clone 6D5, 1:300, catalog 115530, RRID:AB_830707), αCD4-BV650 (clone RM4-5, 1:600, catalog 100555, RRID:AB_2562529), αCD150-PE (clone TC15-12.F12.2, 1:200, catalog 115903, RRID:AB_313682), αCD48–Alexa Fluor 700 (clone HM48-1, 1:100, catalog 103426, RRID:AB_10612755), αCD16/32-PE-Cy7 (clone 93, 1:400, catalog 101307, RRID:AB_312806), αCD4-PE-Cy7 (clone GK1.5, 1:600, catalog 100421, RRID:AB_312706), αCD8–Alexa Fluor 700 (clone 53-6.7, 1:800, catalog 100729, RRID:AB_493702), αCD4-APC-Cy7 (clone GK1.5, 1:600, catalog 100414, RRID:AB_312699), αCD8-PE-Cy7 (clone 53-6.7, 1:600, catalog 100721, RRID:AB_312760), αCD25-PerCP-Cy5.5 (clone PC61, 1:300, catalog 102030, RRID:AB_893288), αHelios–Alexa Fluor 647 (clone 22F56, 1:40, catalog 137208, RRID:AB_10552902), Armenian hamster IgG–Alexa Fluor 647 (clone HTK888; 1:1667), αCD8-PerCP-Cy5.5 (clone 53-6.7, 1:600, catalog 100734, RRID:AB_2075238), αCD62L-PE (clone MEL-14; 1:800, catalog 104407, RRID:AB_313094), αCD62L–Pacific Blue (clone MEL-14; 1:200, catalog 104423, RRID:AB_493381), αCD44-APC-Cy7 (clone IM7; 1:200), αI-A/I-E-APC-Cy7 (clone M5/114.15.2; 1:200, catalog 103028, RRID:AB_830785), rat IgG2b κ-APC-Cy7 (clone RTK4530; 1:200, catalog 400624, RRID:AB_326566), annexin V–Alexa Fluor 647 (1:100, catalog 640911), annexin V–PE (1:200, catalog 640908), αCD8-PE (clone 53-6.7, 1:600, catalog 100708, RRID:AB_312747), αLy-6C/G-APC (clone RB6-8C5, 1:200, catalog 108412, RRID:AB_313377), αCTLA-4–BV605 (clone UC10-4B9, 1:50, catalog 106323, RRID:AB_2566467), Armenian hamster IgG-BV605 (clone HTK888; 1:50, catalog 400943), αTGF-β1–PE (clone TW7-16B4, 1:200, catalog 141403, RRID:AB_10730610) and IgG1 κ–PE (clone MOPC-21, catalog 400113, RRID:AB_326435), αTNFRSF4-BV421 (clone OX-86, 1:50, catalog 119411, RRID:AB_10962569), rat IgG1 κ–BV421 (clone RTK2071, 1:50, catalog 400429, RRID:AB_10900998), αTIGIT-PE-Dazzle (clone 1G9, 1:100, catalog 142111, RRID:AB_2687311), rat IgG1 κ–PE-Dazzle (clone MOPC-21, 1:100, catalog 400157, RRID:AB_10897939), and αLy-6A/E-APC (clone D7; 1:100, catalog 108111, RRID:AB_313348) were purchased from BioLegend. αCD8a-BUV395 (clone 53-6.7, 1:600, catalog 563786, RRID:AB 2732919) and αCD117-BUV395 (clone 2B8, 1:300, catalog 564011, RRID:AB_2738541) were purchased from BD Biosciences. αCD34–eFluor 450 (clone RAM34; 1:100, catalog 48-0341-82, RRID:AB_2043837), αKi-67–PE (clone SolA15, 1:100, catalog 14-5698-82, RRID:AB_10854564), rat IgG2a κ–PE-Cy7 (clone eBR2a; 1:100), αGITR-PE-Cy7 (clone DTA-1, 1:400, catalog 25-5874-80, RRID:AB_10544396), αCD30-PE (clone mCD30.1, 1:10, catalog 12-0301-81, RRID:AB_465628), Armenian hamster IgG (clone eBio299Arm, 1:10, catalog 12-4888-83, RRID:AB_470074), viability dye e450 (1:4000), αMHC I (clone 28-14-8, 1:100, catalog: 16-5999-82, RRID: AB_469197), rat IgG2b κ isotype control (clone eBM2a, 1:100, catalog 16-4724-82, RRID: AB_470164), and viability dye e506 (1:1000) were purchased from Thermo Fisher Scientific. Lin^+^ cells were excluded by magnetic-activated cell sorting (MACS) using biotinylated αCD19 (clone 6D5, 1:300, catalog 115504, RRID:AB_313639), αCD3e (clone 145-2C11, 1:300, catalog 100304, RRID:AB_312669), αLy-6G/C (clone RB6-8C5, 1:300, catalog 108404, RRID:AB_313369), and αTer119 (clone Ter-119; 1:300, catalog 116203, RRID:AB_313704) from BioLegend, followed by a second staining step with streptavidin Horizon-V500 (1:1000, catalog 561419, RRID:AB_10611863) from BD Biosciences after the separation.

Human antibodies were used. αCD34-APC (clone 561, 1:80, catalog 343607, RRID:AB_2074356), αCD34-APC-Cy7 (clone 561, 1:100, catalog 343614, RRID:AB_2571927), αCD45–Pacific Blue (clone HI30, 1:300, catalog 304029, RRID:AB_2174123), αCD38-PE-Cy7 (HIT2, 1:50, catalog 303522, RRID:AB_893314), αCD90-PerCP-Cy5.5 (clone 5E10, 1:100, catalog 328117, RRID:AB_961312), αCD3-BV786 (clone OKT3, 1:100, catalog 317329, RRID:AB_11219196), aCD4-PE-Cy7 (clone OKT4, 1:50, catalog 317414, RRID:AB_571959), αCD25-AF700 (clone BC96, 1:200, catalog 302622, RRID:AB_493755), αCD8a-PerCP-Cy5.5 (clone CD8, 1:100, catalog 300923, RRID:AB_1575079), αTNFRSF4-PE (clone Ber-ACT35, 1:30, catalog 350003, RRID:AB_10641708), and IgG1 κ–PE (clone MOPC-21, catalog 400113, RRID:AB_326435) were from BioLegend. αCD127-BUV737 (clone HIL-7R-M21, 1:50, catalog 612795, RRID:AB_2870122) was from BD Biosciences.

Lin^+^ cells were excluded by staining using biotinylated αCD2 (clone RPA-2.10, 1:100, 300204, RRID:AB_314028), αCD3 (clone OKT3, 1:100, catalog 317320, RRID:AB_10916519), αCD14 (clone HCD14, 1:100, catalog 325624, RRID:AB_2074052), αCD16 (clone 3G8, 1:100, catalog 302004, RRID:AB_314204), αCD19 (clone HIB19, 1:100, catalog 302204, RRID:AB_314234), αCD56 (clone HCD56, 1:100, catalog 318320, RRID:AB_893390), and αCD235ab (clone HIR2, 1:100, catalog 306618, RRID:AB_2565773) (all from BioLegend), followed by a second step using streptavidin Horizon-V500 (1:1000, BD Pharmingen, catalog 561419, RRID:AB_10611863).

Flow cytometric analysis on BM and lin^–^ BM cells, blood cells, and splenocytes were performed following RBC lysis. Samples were analyzed on a BD Fortessa, and sorting procedures were performed using a BD FACS Aria III (BD Pharmingen). Data were collected using FACSDiva software (BD Pharmingen) analyzed using FlowJo software (Tree Star Inc.). Effective separation after sorting by FACS was assessed by reanalyzing a fraction of the sorted samples by flow-cytometry analysis (purity after FACS-sorting: 96.2 ± 1.8%).

### Patient samples

BM aspirates from untreated, newly diagnosed CML patients at the Department of Hematology and Central Hematology Laboratory were obtained between 2015 and 2020. Patient characteristics are listed in Supplemental Table 2. Patient data were collected and managed using REDCap electronic data capture tools hosted at the DBMR (62).

### Mice

BL/6 mice were purchased from Charles River Laboratories, and *Foxp3*^DTR/GFP^ mice were obtained from the Jackson Laboratory (33). MHC class II^–/–^ (*H2*^–/–^) mice were received from the Swiss Immunological Mouse Repository (63). Perforin^–/–^ (*Prf*^–/–^) mice were provided by P. Krebs (Institute of Pathology, University of Bern; ref. 64). Experiments were performed with age-matched (6–8 weeks) and sex-matched animals of both sexes. Mice were housed under specific pathogen–free conditions in individually ventilated cages with food and water ad libitum, and they were regularly monitored for pathogens. Mice were assigned to different treatment groups through randomization, and all experiments were conducted and analyzed in a nonblinded fashion.

### Colony-forming assays

#### Mouse.

In total, 5 × 10^3^ MACS-purified lin^–^ cells were plated in semisolid methylcellulose, as previously described (19). GFP^+^ colonies were determined after 7 days with an inverted fluorescence microscope.

For in vitro coculture experiments, 1 × 10^3^ FACS-purified LSCs were incubated with perforin-deficient or -proficient CD8^+^ T cells from BM of CML mice at a ratio of 1:1 overnight in RPMI supplemented with 10% FCS (Thermo Fisher Scientific), 1% penicillin-streptomycin (MilliporeSigma), 1% L-glutamine (MilliporeSigma), SCF (100 ng/mL) and TPO (20 ng/mL) (Miltenyi Biotec), followed by plating in methylcellulose. Alternatively, LSCs pretreated with the granzyme B inhibitor I (100 μM, MilliporeSigma) for 1 hour at 37°C were coincubated with CD8^+^ T cells from BM of CML mice at a ratio of 1:1 overnight, followed by plating in methylcellulose. In addition, LSCs were cocultured overnight with CD8^+^ T cells and Tregs pretreated for 2 hours with an Tnfrsf4 antibody (clone OX-86, 30 μg/mL, BioXCell, catalog BE0031, RRID:AB_1107592) or control IgG1 antibody (catalog BE0088, RRID:AB_1107775) at a ratio of 1:1:1 in triplicate followed by plating in methylcellulose. For each round of serial colony replating, total cells were collected from the methylcellulose, and 1 × 10^4^ cells were replated into methylcellulose without any T cells. Colony numbers were assessed with inverted light microscopy after 7 days for each round of plating (≥ 30 cells/colony).

#### Human.

In total, 1 × 10^3^ FACS-purified CD34^+^CD38^–^ CML were plated in semisolid methylcellulose as previously described (19) (Supplemental Table 2). Colonies were determined after 14 days with an inverted light microscope. For coculture experiments, 1 × 10^3^ FACS-purified CD34^+^CD38^–^ CML stem/progenitor cells (CML also pretreated with the granzyme B inhibitor I) were coincubated with BM CD8^+^ T cells and/or CD4^+^CD127^lo^CD25^+^ Tregs at a ratio of 1:1:1 followed by plating in methylcellulose. For each round of serial colony replating, total cells were collected from the methylcellulose, and 1 × 10^4^ cells were replated into methylcellulose without any T cells. Colony numbers were assessed with inverted light microscopy after 14 days for each round of plating (≥ 30 cells/colony).

### Leukemia mouse models

Chronic phase CML was induced and monitored as described before (31). Briefly, FACS-purified LSKs from the BM of donor mice were transduced twice on 2 consecutive days with a BCR-ABL1-GFP retrovirus by spin infection. In total, 3 × 10^4^ cells were injected i.v. into the tail vein of nonirradiated syngeneic recipients.

Blast crisis CML was induced as previously described (65). FACS-purified LSKs were simultaneously transduced with BCR-ABL1-CFP and NUP98-HOX-GFP retrovirus in a RetroNectin precoated plate on 2 consecutive days. After 2 transduction rounds, NUP98-HOX-GFP/BCR-ABL1-CFP–double-positive cells were FACS purified and injected into sublethally irradiated recipients (4.5 Gy) to expand the leukemic cells. In total, 5 × 10^3^ NUP98-HOX-GFP/BCR-ABL1-CFP–double-positive cells from primary recipient mice were injected i.v. into the tail vein of nonirradiated syngeneic recipients.

For Treg depletion, DT (15 ng/g, MilliporeSigma) was administered i.p. at different days indicated in the figure legends. Sterile PBS (MilliporeSigma) was used as a control treatment. To deplete CD8α and CD8β T cells, mice were treated with 75 μg murine αCD8α mAb (clone 53-6.7, BioXCell, catalog BE0004-1, RRID:AB_1107671) i.p. at different days indicated in the figure legends. To compare LSC activity in vivo, 5 × 10^6^ whole BM (WBM) cells from primary CML mice were injected i.v. into lethally irradiated (6.5 Gy twice with 4 hours interval) secondary recipient mice.

For Tnfrsf4 antibody treatment experiments, Tnfrsf4 antibody (200 μg/mouse, clone OX-86, BioXCell, catalog BE0031, RRID:AB_1107592) was administered i.p. 6 times every second day, starting at day 12. Rat IgG1 κ (BioXCell, catalog BE0088, RRID:AB_1107775) was used as a control treatment.

### LSC analysis

The LSC numbers in chronic phase and blast-crisis CML mice were analyzed phenotypically by FACS analysis as previously described (18, 19). Briefly, LSC subpopulations in BCR-ABL1-GFP^+^ lin^–^ BM cells were defined as follows: L-HPC-1 (Sca-1^+^c-kit^hi^CD48^+^CD150^–^), L-HPC-2 (Sca-1^+^c-kit^hi^CD48^+^CD150^+^), L-MPPs (Sca-1^+^c-kit^hi^CD48^–^CD150^–^), and LT-LSCs (Sca-1^+^c-kit^hi^CD48^–^CD150^+^). For blast-crisis CML, the disease-initiating cells were defined as NUP98-HOX-GFP^+^BCR-ABL1-CFP^+^lin^–^Sca-1^+^c-kit^hi^CD135^+^CD150^–^ (66).

### Ki-67 staining

Ki-67 staining was performed with Foxp3/Transcription Factor Staining Buffer Set (Thermo Fisher Scientific) according to manufacturer’s protocol. After surface marker staining, cells were incubated in fixation/permeabilization working solution for up to 18 hours at 4°C, followed by washing with permeabilization buffer and intracellular staining with Ki-67 PE antibody for 30 minutes at 4°C.

#### High-throughput transcriptome analysis using next-generation RNA-Seq.

Total RNA was extracted from Tregs derived from the BM of naive and CML-bearing *Foxp3*^DTR/GFP^ mice (*n =* 3/group) using the RNeasy Micro Kit (catalog 74004, Qiagen). Total RNA quality was determined by a Bioanalyzer using the RNA 6000 Nano Chip (Agilent Technologies) and quantified by fluorometry using the Quantifluor RNA System Kit (catalog E3310, Promega) on a Quantus Fluorometer Instrument (Promega).

Library preparation was performed from total RNA using the SMART-Seq v4 Ultra Low Input RNA Kit for Sequencing (Takara Bio). Libraries were quality checked on the Fragment Analyzer using the High Sensitivity NGS Fragment Analysis Kit (Agilent). Samples were pooled to equal molarity, and the pool was quantified by fluorometry, in order to be loaded at a final concentration of 2 pM on the NextSeq 500 instrument (Illumina). Samples were sequenced SR76 using the NextSeq 500 High Output Kit 75-cycles (Illumina), and primary data analysis was performed using the Illumina RTA version 2.4.11 and bcl2fastq v2.20.0.422.

### RNA-Seq data analysis

The RNA-Seq data was assembled by SeqMan NGen software v.15 and analyzed using ArrayStar software v.15 (DNASTAR). The software allows statistical analyses of differential gene expression using EdgeR or DESeq2. For our analysis, we used DEseq2. The level of gene expression was assessed after normalization and log_2_ transformation. The data set was analyzed by 2-way ANOVA. Genes with significant difference in their expression at an FDR *P* value less than 0.05, and fold differences ≥ 1.5 were selected. Data were clustered using standard Euclidean’s method based on the average linkage, and heatmaps were generated according to the standard normal distribution of the values.

### GO analysis

GO enrichment was assessed using Partek Genomics Suite software, v.7 (Partek). The list of differently expressed genes was grouped into functional hierarchies. Enrichment scores were calculated using a χ^2^ test comparing the proportion of the gene list in a group to the proportion of the background genes. A value of 3 or higher corresponded to a significant overexpression (*P <* 0.05).

### Quantitative PCR

For quantitative PCR (qPCR), total RNA was extracted using the Quick-RNA MiniPrep kit (Zymo Research). Complementary DNA synthesis was performed using 2.5 × 10^–4^ units/μL hexanucleotide mix (Roche), 0.4 mM deoxynucleotide mix (MilliporeSigma), 1.25 units/μL RNasin, and 4 units/μL reverse transcriptase (Promega). Gene expression analysis was accomplished for murine *Gzma* and *Gzmb* using self-designed primers and SYBR green reaction (Roche; *Gzma,* FV: 5′ CACTGTAACGTGGGAAAGAG 3′, RV: 5′ GTGAAGGATAGCCACATTTCTG 3′; *Gzmb,* FV: 5′ CTGCTAAAGCTGAAGAGTAAGG 3′, RV: 5′ GCTCAACCTCTTGTAGCGT 3′). Samples were measured in duplicate or triplicate, including nontemplate controls using a QuantStudio 3 Real-Time PCR system (Applied Biosystems). Relative quantification of gene expression was normalized against a reference gene (*Gapdh or* ACTB) and calculated as an exponent of 2 (2^ΔCt^).

### IHC

#### Human.

To study the distribution of FOXP3^+^ Tregs and their spatial proximity to CD34^+^ and CD8^+^ cells, formalin-fixed, paraffin-embedded (FFPE) tissues from 10 CML and 4 control BM core biopsies were analyzed. Sections were cut to 2 μm thickness, and IHC double stainings of full slides were performed for both FOXP3/CD8 and FOXP3/CD34 (anti–human FOXP3, eBioscience, 1:200, catalog 14-4777-80, RRID:AB_467555; anti–human CD8, 1:100, catalog M7103, RRID:AB_2075537; and anti–human CD34, 1:50, Cell Marque, catalog 134M-16, RRID:AB_1159227) using a Leica BOND RX automated immunostainer (Leica Biosystems). A counting field of 1.2 mm^2^ was randomly selected, and FOXP3^+^ Tregs were counted at 20× magnification in CML and control BM biopsies (healthy donors). Close proximity between FOXP3^+^ Tregs and CD8^+^ or CD34^+^ LSPCs was defined as a distance of less than or equal to 2 cell nuclei. Since FOXP3^+^ Tregs were observed at a low frequency in control BM biopsies, and to enable a sufficient comparison between control BM and CML biopsies, control BM biopsies with less than 5 FOXP3^+^ Tregs (counted in 1.2 mm^2^) were additionally screened longitudinally for additional Tregs that could be included into the final analysis.

#### Mouse.

The distribution of FOXP3^+^ Tregs and their spatial proximity to CD8^+^ cells were analyzed using FFPE tissues from 8 CML and 9 control murine BM core biopsies. Sections were cut to 2.5 μm thickness, and IHC double stainings of full slides were performed for FOXP3/CD8 (rat anti–mouse FOXP3, clone FJK-16s, eBioscience, 1:00, catalog 14-5773-80, RRID:AB_467576; rat anti–mouse CD8, clone 4SM15, 1:100, eBioscience catalog 14-0808-80, RRID:AB_2572861) using a Ventana Discovery ULTRA automated immunostainer (Roche Diagnostics). A counting field of 1.2 mm^2^ was randomly selected, and FOXP3^+^ Tregs were counted at 20× magnification in CML and control BM biopsies. Close proximity between FOXP3^+^ Tregs and CD8^+^ cells was defined as a distance of less than or equal to 2 cell nuclei.

To illustrate the distribution pattern of FOXP3^+^ cells in human and murine control and CML BM core biopsies, whole slides were analyzed using QuPath (software version 0.1.2; ref. 67).

### Data and code availability

All RNA-seq data compiled for this study are made publicly available on the Gene Expression Omnibus (GEO) website (http://www.ncbi.nlm.nih.gov/geo/) under the accession number GSE174190. This study does not include the development of new code.

### Statistics

Statistical analysis was performed using GraphPad Prism 7.04 (GraphPad Software). Statistical tests applied to determine significance for each experiment are detailed in the corresponding figure legend. Data are represented as mean ± SEM and assumed to distribute normally. For Treg depletion experiments, leukemia load was determined in the blood when disease was established, and mice were randomized using GraphPad software random number generator to the different treatment groups based on disease burden. Data were analyzed using Student’s *t* test (2-tailed), 1-way ANOVA followed by Tukey’s or Dunnett’s post hoc test (2-sided), and 2-way ANOVA followed by Sidak’s post hoc test (2-sided). Significant differences in Kaplan-Meier survival curves were determined using the log-rank test. Human data from the microarray data set were checked with column statistics for normal distribution and analyzed with Student’s *t* test (2-tailed). Correlations were determined using Spearman correlations (2-sided). All *P* values were considered as significant when *P <* 0.05. All experiments were at least performed twice in independent experiments.

### Study approval

Animal experiments were approved by the local experimental animal committee of the Canton of Bern and performed according to Swiss laws for animal protection (KEK 75/17, 78/17, BE56/20, and BE59/20).

Analysis of BM samples was approved by the local ethical committee of the Canton of Bern, Switzerland (KEK 122/14 and 2019-01627). Written informed consent was collected from all patients who donated BM.

## Author contributions

MH designed and performed experiments, analyzed and interpreted data, and contributed to the preparation and writing of the manuscript. VR designed experiments, performed experiments, and analyzed and interpreted data. CS, SF, RR, and NS designed and performed experiments and analyzed data. GMB collected and contributed CML patient samples and interpreted data. AFO interpreted data, designed experiments, and revised the manuscript. CR designed and supervised the study, interpreted data, and wrote the manuscript. All authors revised the manuscript and approved its final version.

## Supplementary Material

Supplemental data

Supplemental table 1

## Version 1 11/02/2021

In-Press Preview

## Version 2 12/08/2021

Electronic publication
