# Supplementary material for: Tnfrsf4-expressing regulatory T cells promote immune escape of chronic myeloid leukemia stem cells
Source: JCI Insight. 2021 Dec 8;6(23):e151797. doi: 10.1172/jci.insight.151797 (PMC8675189; doi:10.1172/jci.insight.151797)
Supplement: Supplemental data [file jciinsight-6-151797-s157.pdf]

Supplemental information

Supplementary Table 1: List of differentially expressed genes between Treg derived from BM of naïve and CML mice. Data are provided in form of an Excel Spreadsheet.

Supplementary Table 2: CML patient characteristics.

Age, blood leukocyte counts, the percentage of BM cellularity and the percentage of BM blasts at the time of diagnosis, as well as the type of BCR-ABL1 translocation are shown for CML patients (ID 1-7). Abbreviations: n.d., not determined;

**ID**	**Sex**	**Age**	**Blood leukocytes [x10^9/l]**	**BM cellularity [%]**	**BM blasts [%]**	**BCR-ABL1 type**	**BCR-ABL1 mutation**
CML 1	f	42	106.2	90	< 2	b2a2	n.d.
CML 2	m	58	242	100	< 2	b3a2	n.d.
CML 3	m	84	39.5	95	< 5	b3a2	n.d.
CML 4	m	40	254	100	<5	b2a2, b3a2	n.d.
CML 5	m	42	167	hypercellula r	>5	e6a2	n.d.
CML 6	m	36	108	95	<2	b3a2	n.d.
CML 7	f	72	85	95	<2	b3a2	n.d.
HD 1	f	65	-	-	-	-	-
HD 2	f	86	-	-	-	-	-
HD 3	f	70	-	-	-	-	-
HD 4	m	28	-	-	-	-	-
HD 5	f	70	-	-	-	-	-
HD 6	f	68	-	-	-	-	-

BM blasts (defined as $CD34^+$ and/or c-kit^+): < 5%

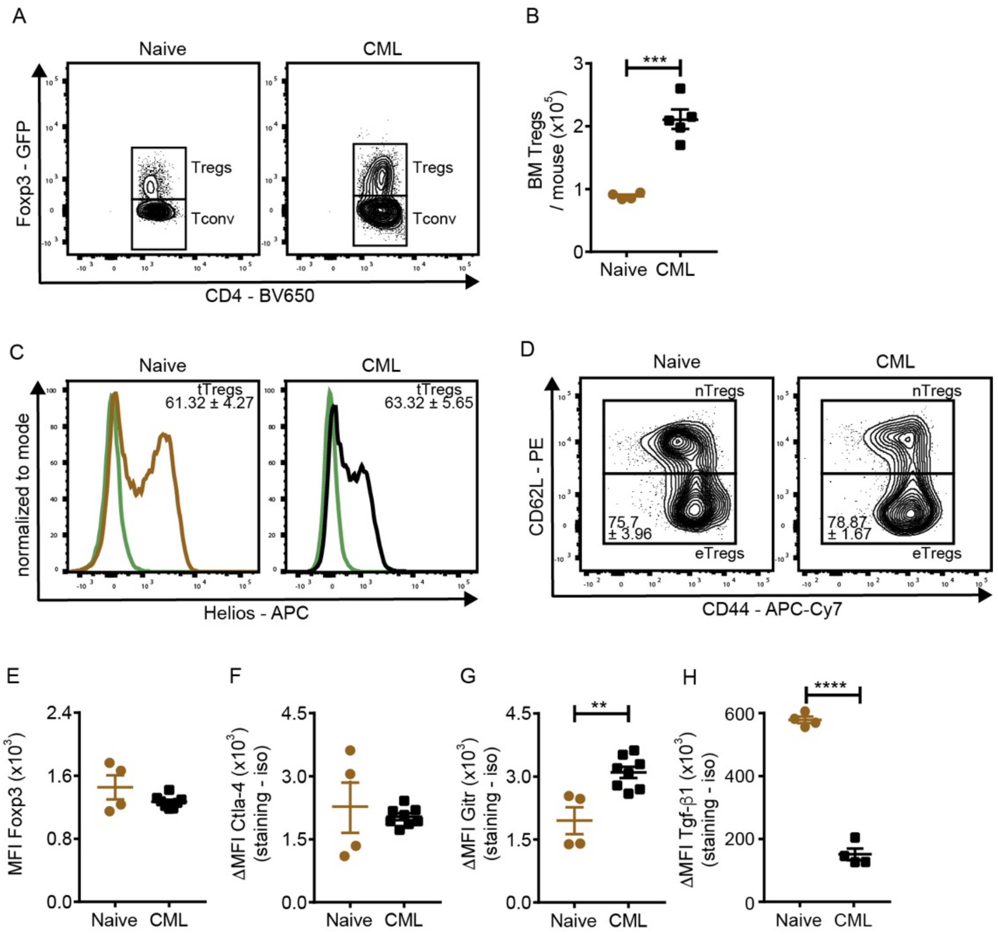

Fig. S1: Gating scheme for Tregs and phenotypic analysis of Tregs from the spleen of naïve and CML mice. (A) Gating strategy of Foxp3^{+} Tregs, pre-gated on CD4^{+} T cells. **(B)** Absolute numbers of Tregs in naïve and CML BM (Naive: n=4 mice; CML: n=5 mice); t-test **(C)** Representative histogram for Helios^{+} thymic-derived Tregs (tTregs) and Helios^{-} peripheral-induced Tregs (pTregs) in the spleen of naïve (n=11) and CML *Foxp*3^{DTR} mice (n=8). Cells are pre-gated on CD4^{+} Foxp3-GFP^{+} Tregs. Staining: beige (naïve) and black (CML); isotype: green; t-test. (**D**) Representative Zebra-Plot for CD62L^{+}CD44^{low} naïve/resting Tregs (nTregs) and CD62L^{-}CD44^{high} effector Tregs (eTregs) in the spleen of naïve (n=5) and CML *Foxp3*^{DTR} mice (n=5); t-test. (**E**) MFI Foxp3-GFP^{+} expression (Foxp3) of CD4^{+} Foxp3-GFP^{+} Tregs in the spleen of naïve (n=4) and CML *Foxp3*^{DTR} mice (n=8); t-test. **(F)** ΔMFI of CTLA-4 on CD4^{+} Foxp3-GFP^{+} Tregs in the BM naïve (n=4) and CML *Foxp3*^{DTR} mice (n=8); t-test. ΔMFI=staining-isotype. (**G**) ΔMFI of GITR on CD4^{+} Foxp3-GFP^{+} Tregs in the spleen of naïve (n=4) and CML *Foxp3*^{DTR} mice (n=8); t-test. ΔMFI =staining-isotype. (**H**) ΔMFI of TGF-β1

on $CD4^+$ Foxp3-GFP^+ Tregs in the spleen of naïve (n=4 mice) and $\textit{Foxp3}^{DTR}$ CML mice (n=4 mice); t-test. ΔMFI =staining-isotype. Data are displayed as mean ± SEM. Statistics: ** $P<0.01$, *** $P<0.001$, **** $P<0.0001$, Related to Figure 1.

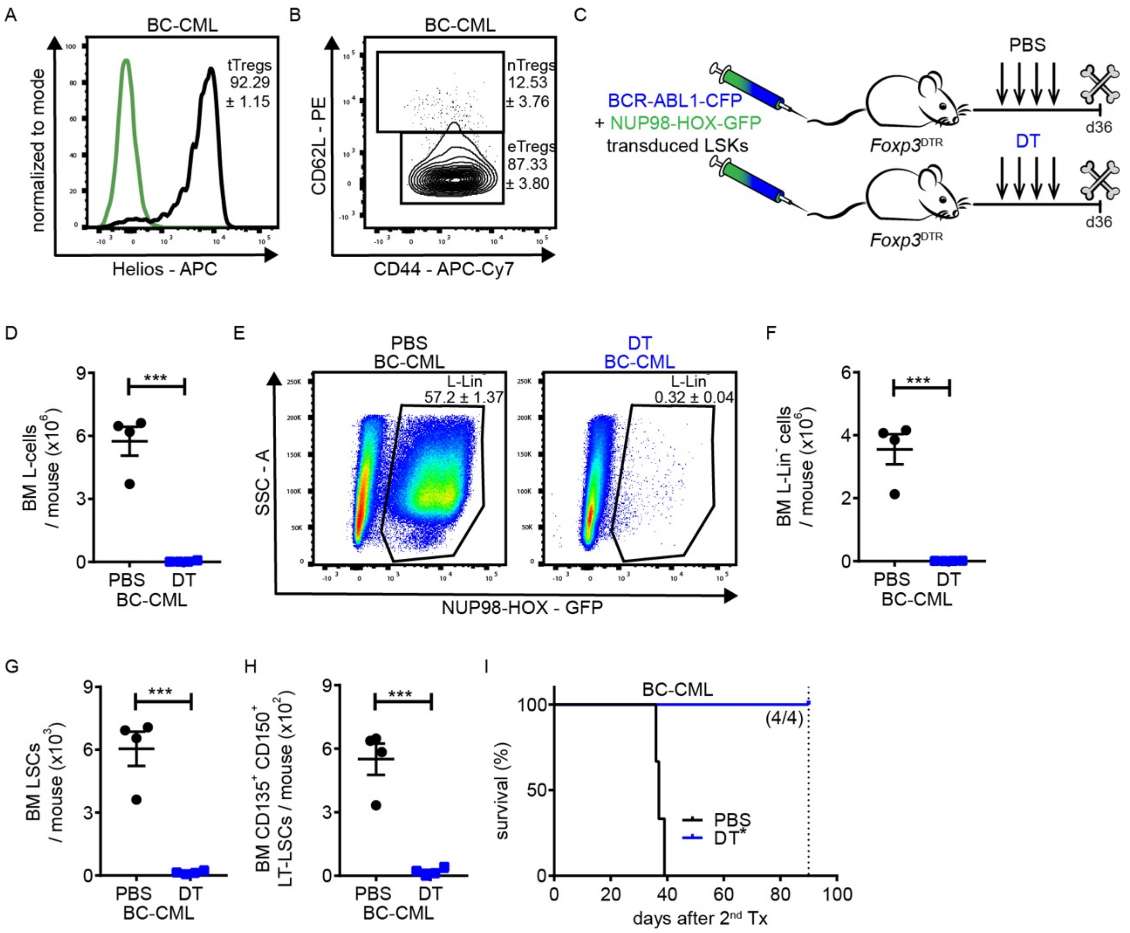

Fig. S2: BM tTregs contribute to disease development in BC-CML. (A to I) LSKs of BL/6 mice were co-transduced with BCR-ABL1-CFP and NUP98-HOX-GFP retroviruses and BCR-ABL1-CFP/NUP98-HOX-GFP^{+} double-positive cells were injected into non-irradiated *Foxp3*^{DTR} recipient mice (BC-CML). **(A)** Frequencies of Helios^{+} thymic-derived Tregs (tTregs), (**B**) CD44^{low}CD62L^{high} resting/naïve Tregs (nTregs) and CD44^{high}CD62L^{-} effector Tregs (eTregs) in the BM of BC-CML mice. Cells are pre-gated on CD4^{+} Foxp3^{+} Treg cells (n=6 mice/group). **(C to H)** Experimental setup. BC-CML mice were randomized and subjected to treatment with DT or PBS as a control on days 7, 8, 13 and 14 after BC-CML initiation. BC-CML mice were sacrificed at day 36 and BM was analyzed (n=4 mice/group). **(D)** Number of BM L-cells (n=4 mice/group); t-test. **(E)** Frequencies and **(F)** absolute numbers of L-lin^{-} cells (n=4 mice/group); t-test. **(G)** Absolute number of BC-CML Sca-1^{+}c-kit^{high} LSCs and (**H**) CD135^{+}CD150^{-} LT-LSCs (n=4 mice/group); t-test. (**I**) BM cells of primary CML mice (day 36)

were injected i.v. into lethally irradiated secondary BL/6 recipients and survival was monitored (n=4 mice/group); log-rank test. Dotted line represents the time point experiment termination at day 90. Data are displayed as mean±SEM. ** $P<0.01$, *** $P<0.001$ and **** $P<0.0001$. Related to Figure 3.

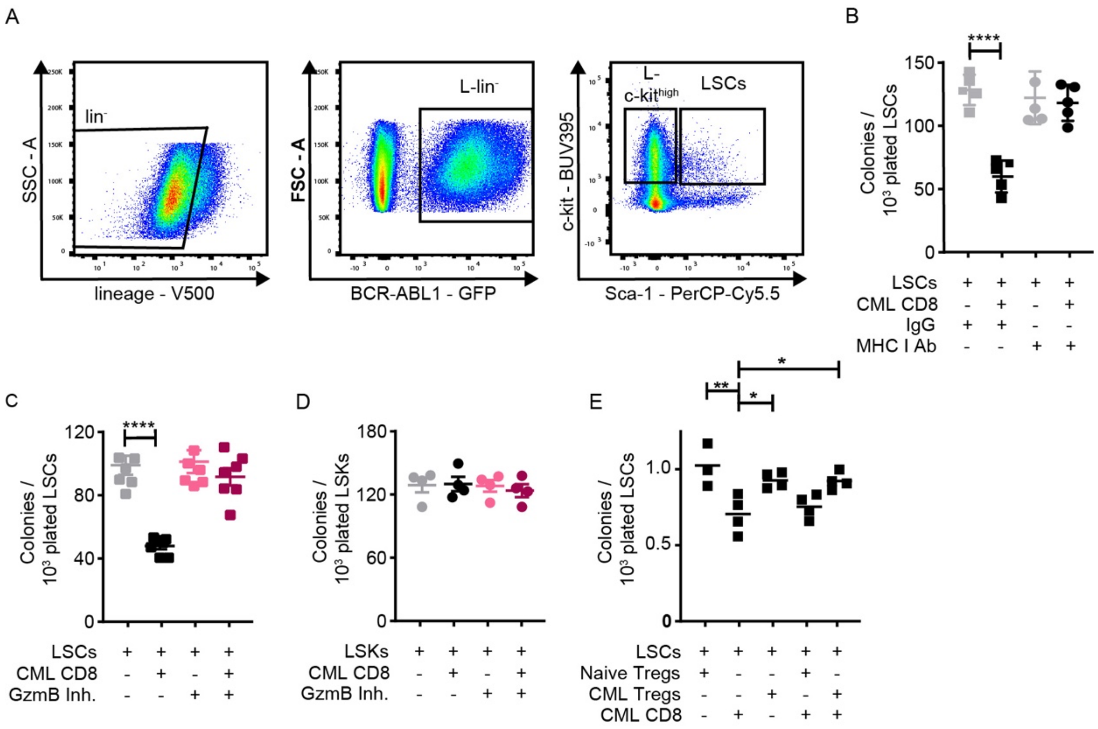

Fig. S3: CD8$^+$ T cells from the BM of CML mice eradicate LSCs but not HSCs by perforin- and granzyme B-mediated lysis. (A) Gating strategy used to identify leukemic lin$^-$ (L-lin$^-$) cells, L-c-kit^{high} cells and LSCs of MACS-purified lin$^-$ BM cells. **(B)** LSCs pre-incubated for 1h in the presence of anti-MHC I antibody (clone: 28-14-8; 10 µg/ml) or control IgG prior to overnight co-culture with BM CD8$^+$ T cells from CML-bearing BL/6 mice (ratio 1:1) were plated in duplicates in methylcellulose and colony formation was assessed 7 days later. Numbers of colonies per 10^3 plated LSCs from one out two independent experiments are shown; t-test (n=5 mice/group). **(C)** LSCs pre-incubated in the presence and absence of the Granzyme B Inhibitor I prior to overnight co-culture with BM CD8$^+$ T cells from CML-bearing BL/6 mice (ratio 1:1) were plated in duplicates in methylcellulose and colony formation was assessed 7 days later. Numbers of colonies per 10^3 plated LSCs pooled from two independent experiments are shown; t-test (n=7 mice/group). (**D)** HSCs from naïve BL/6 mice pre-incubated in the presence and absence of the Granzyme B Inhibitor I prior to overnight co-culture with BM CD8$^+$ T cells from CML-bearing BL/6 mice (ratio 1:1) were plated in duplicates in methylcellulose and colony formation was assessed 7 days later. Numbers of colonies per 10^3

plated LSKs is shown; t-test (n=4 mice/group). **(E)** LSCs (pooled from n= 4 CML mice) were pre-incubated overnight in duplicates in presence and absence of BM CD8$^+$ T cells and BM Tregs from CML-bearing BL/6 mice (ratio 1:1:1, n=4) or BM Tregs from naïve BL/6 mice (n=3) followed by plating in methylcellulose. Colony formation was assessed 7 days later; One-Way ANOVA followed by Dunnett's post-test (two-sided). Data are represented as mean ± SEM or mean only. * *P*<0.05, ** *P*<0.01. Related to Figures 4 and 5.

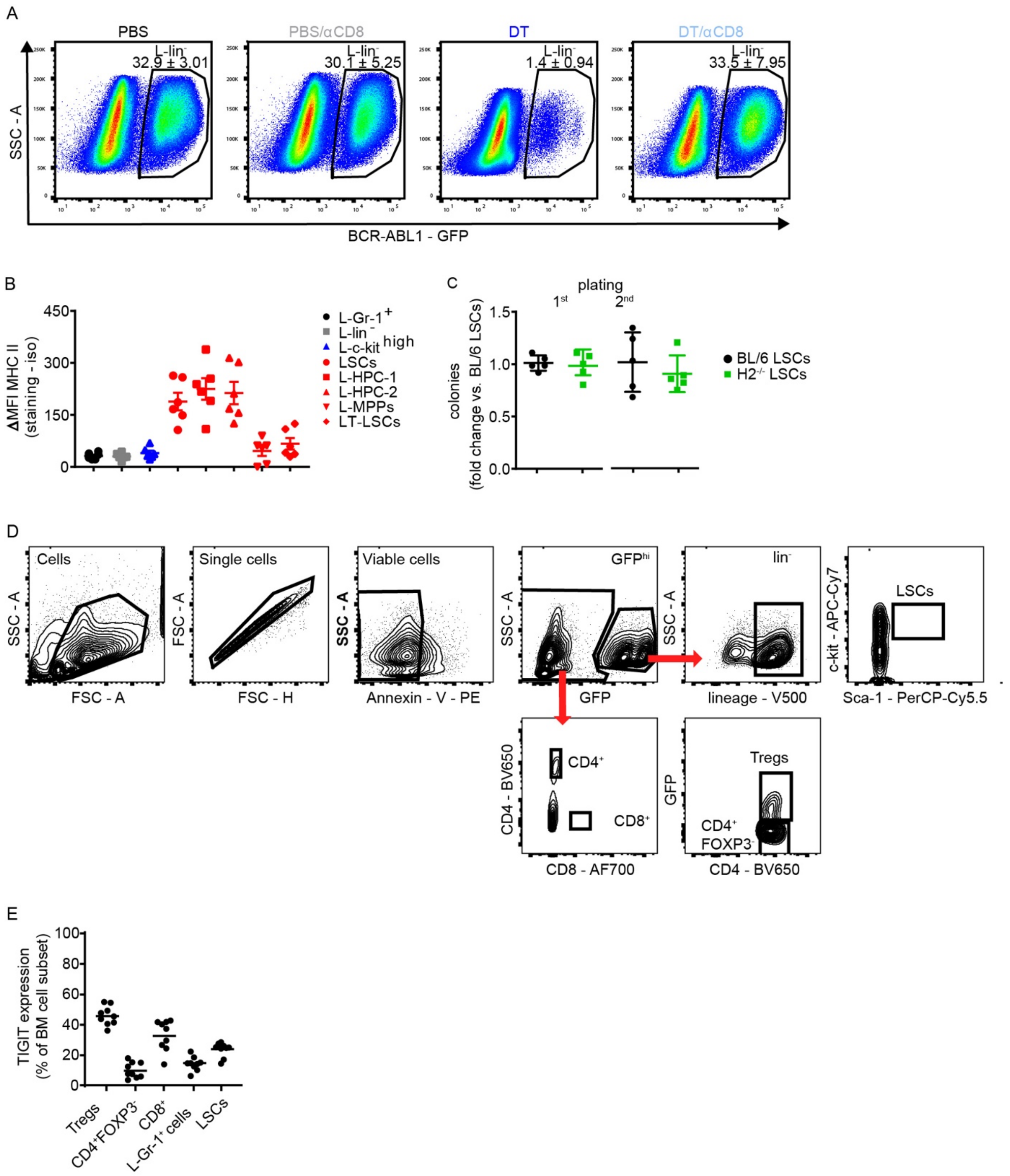

Fig. S4: Representative FACS plots of L-lin$^-$ cell frequencies after treatment with DT or αCD8 mAb, MHC class II expression on LSCs and Tigit expression on Tregs, CD8$^+$ T cells, CD4$^+$ Foxp3$^-$ T cells and leukemia cells in the BM of CML mice. (A) Experimental setup. BCR-ABL1-GFP-transduced LSKs were injected intravenously into non-irradiated *Foxp3*^{DTR} recipients. After establishment of the disease (day 13), mice were randomized to PBS/DT and αCD8 mAb treatment. DT was administered i.p. at days 13, 14, 19 and 20; αCD8

mAb at days 13 and 15 i.p. Mice were sacrificed at day 21 after transplantation of leukemic cells and the BM for all treatment groups was analyzed for the frequency of L-Lin^{-} cells. (Groups: PBS: n=3 mice, PBS + αCD8: n=4 mice, DT: n=5 mice and DT + αCD8: n=5 mice). **(B)** ΔMFI of MHC II on L-Gr-1^{+}, L-lin^{-} and L-c-kit^{high} cells and LSCs with the subpopulations L-HPC-1, L-HPC-2, L-MPPs and LT-LSCs. (n=6 CML mice). ΔMFI: MFI staining – MFI isotype. (**C)** Clonogenic potential of MHC class II (*H2*) $^{-/-}$ and BL/6 LSCs in vitro. 10^{3} *H2*$^{-/-}$ and BL/6 LSCs were plated in methylcellulose and colony formation capacity was assessed 7 days later (n=5 mice/group). For re-plating experiments 10^{4} cells from primary colony assessed were plated in methylcellulose. **(D)** Gating strategy to identify T cell subsets and LSCs in the BM of CML mice. One representative out of 9 plots is depicted. **(E)** BCR-ABL1-GFP-transduced LSKs were injected intravenously into non-irradiated *Foxp3*^{DTR} recipients (n=9 mice). 14 days later, animals were sacrificed and Tigit expression was determined on CD4^{+} Foxp3-GFP^{+} Tregs CD8^{+} T cells, CD4^{+} Foxp3^{-} T cells, L-Gr-1^{+} and LSCs by FACS. Data are displayed as mean or mean ± SEM. Related to Figures 5-7.

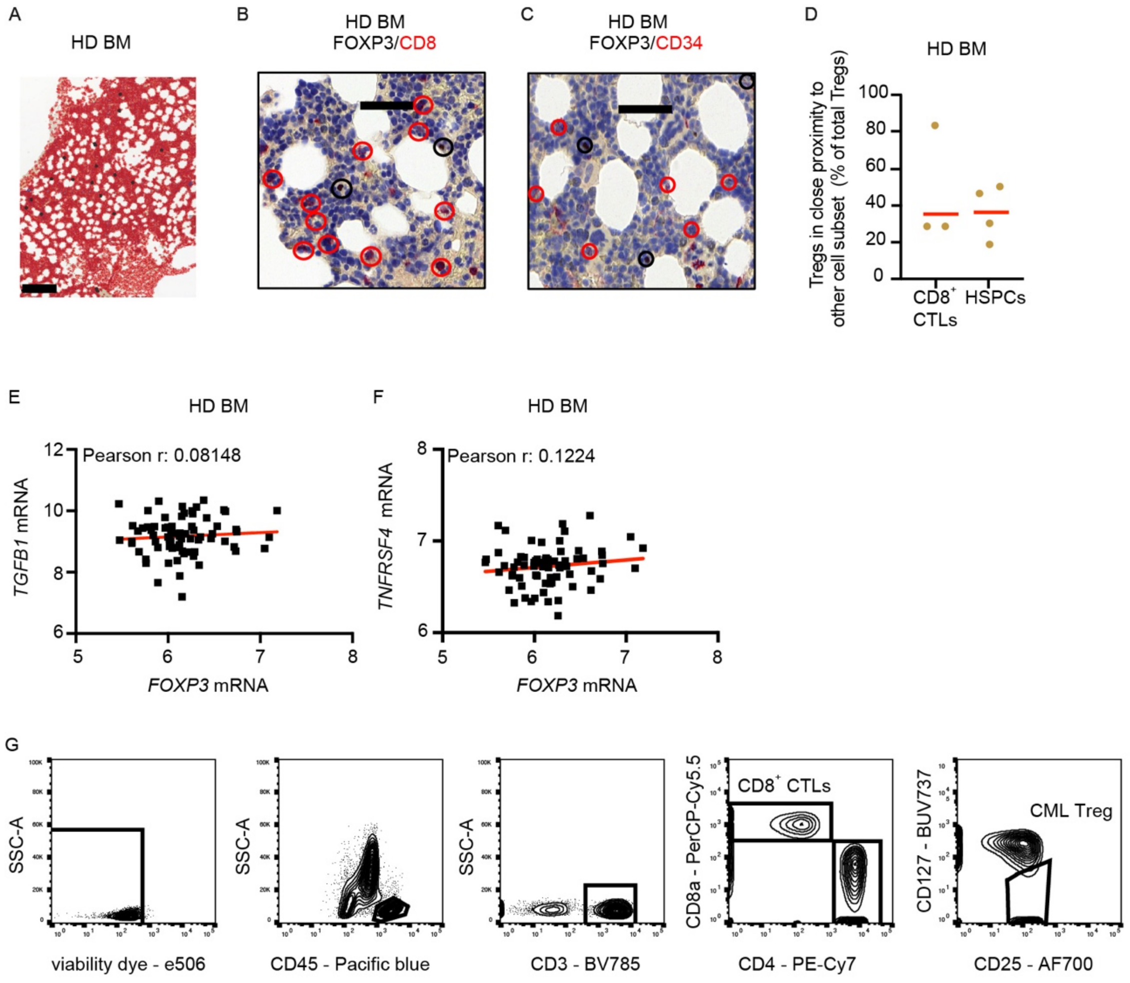

Fig. S5: The majority of Tregs in the BM of CML patients are localized close to CD8^{+} T cells. (A) Representative immunohistochemistry stainings addressing the overall distribution of FOXP3^{+} Tregs in the BM (scale bar 200 μm, n= 10 CML patients). **(B-D)** Representative immunohistochemistry stainings addressing the spatial localization of FOXP3^{+} Tregs in the BM in respect to **(B)** CD8^{+} T cells and **(C)** CD34^{+} hematopoietic stem/progenitor cells (HSPCs, scale bar 50 μm, n= 10 CML patients; FOXP3: brown; CD8^{+} T cells and CD34^{+} HSPCs: red). FOXP3^{+} cells are highlighted with black and CD8^{+} T cells and CD34^{+} HSPCs with red circles. Slides were analyzed with QuPath software. **(D)** Frequency of Tregs located in close proximity to CD8^{+} T cells and LSPCs in the BM of newly diagnosed CML patients (n=10 patients). Slides were analyzed by QuPath software. Close proximity was defined as a distance of ≤ two cell nuclei; t-test. **(E-F)** Correlation of mRNA expression levels of **(E)** *TGFB1* and **(F)** *TNFRSF4* with *FOXP3* in BM of healthy donors (HD: n=73; GSE13159); Spearman correlations. **(G)**

FACS gating strategy to identify $CD8^+$ T cells and Tregs in the BM of CML patients. Data are shown as mean. Related to Figure 8.
